# Cytoplasmic connexin43-microtubule interactions promote glioblastoma stem-like cell maintenance and tumorigenicity

**DOI:** 10.1038/s41419-025-07514-2

**Published:** 2025-05-16

**Authors:** James W. Smyth, Sujuan Guo, Lata Chaunsali, Laurie O’Rourke, Jacob Dahlka, Stacie Deaver, Michael Lunski, Elmar Nurmemmedov, Harald Sontheimer, Zhi Sheng, Robert G. Gourdie, Samy Lamouille

**Affiliations:** 1 Fralin Biomedical Research Institute at Virginia Tech Carilion, Roanoke, VA 24016 USA; 2 Department of Biological Sciences, Virginia Polytechnic Institute and State University, Blacksburg, VA 24061 USA; 3 Virginia Tech Carilion School of Medicine, Roanoke, VA 24016 USA; 4 Department of Neuroscience, University of Virginia School of Medicine, Charlottesville, VA 22903 USA; 5 Carilion Clinic, Roanoke, VA 24016 USA; 6 Scintillon Institute for Biomedical Research, San Diego, CA 92121 USA; 7 Department of Biomedical Engineering and Mechanics, Virginia Polytechnic Institute and State University, Blacksburg, VA 24060 USA

**Keywords:** Cancer stem cells, Biologics

## Abstract

Glioblastoma (GBM) is the most common primary tumor of the central nervous system. One major challenge in GBM treatment is the resistance to chemotherapy and radiotherapy observed in subpopulations of cancer cells, including GBM stem-like cells (GSCs). These cells have the capacity to self-renew and differentiate and as such, GSCs participate in tumor recurrence following treatment. The gap junction protein connexin43 (Cx43) has complex roles in oncogenesis and we have previously demonstrated an association between Cx43 and GBM chemotherapy resistance. Here, we report, for the first time, increased direct interaction between non-junctional Cx43 and microtubules in the cytoplasm of GSCs. We hypothesize that non-junctional Cx43/microtubule complexing is critical for GSC maintenance and survival and sought to specifically disrupt this interaction while maintaining other Cx43 functions, such as gap junction formation. Using a Cx43 mimetic peptide of the carboxyl terminal tubulin-binding domain of Cx43 (JM2), we successfully disrupted Cx43 interaction with microtubules in GSCs. Importantly, administration of JM2 significantly decreased GSC survival in vitro, and limited GSC-derived and GBM patient-derived xenograft tumor growth in vivo. Together, these results identify JM2 as a novel peptide drug to ablate GSCs in GBM treatment.

## Introduction

Glioblastoma (GBM) is a highly malignant and lethal cancer of the central nervous system. The current multimodal therapy for newly diagnosed GBM patients includes surgical resection, radiotherapy, and chemotherapy with temozolomide (TMZ), conferring a median survival of only 14.6 months [1–3], with recent results from phase 3 clinical trials showing an extension up to 20 months, although using more stringent inclusion criteria that limit generalizability [4–6]. Failure to generate more effective treatment strategies is due to the infiltrative nature of GBM tumor cells preventing complete surgical resection [7], and the cellular heterogeneity within GBM tumors, which comprise a sub-population of GBM stem-like cells (GSCs) that are resistant to chemotherapeutic agents including TMZ [8–11]. In fact, GSCs present a high degree of plasticity with the ability to self-renew through symmetric proliferation or differentiate through asymmetric division [12, 13], recapitulating the heterogeneous tumor and overall promoting GBM recurrence [14–16]. Among the signaling pathways that participate in GSC maintenance, Notch signaling is highly active in GSCs, and critical to maintain stem-like properties, promoting self-renewal while suppressing differentiation [17–19]. Notch signaling occurs via activation by single-pass ligand proteins present on the membrane of adjacent cells, with Notch receptors being cleaved and an intracellular domain translocating to the nucleus to activate the transcription of genes including the primary targets of Notch signaling, *HES* and *HEY*. Hes and Hey proteins are transcriptional repressors that, among other functions, contribute to the self-renewal of GSCs [17, 20–22].

Intercellular junctions are crucial to maintaining homeostasis and provide mechanical communication between cells as well as, in the case of gap junctions, direct coupling of neighboring cellular cytoplasms. Dysregulation of these junctions is associated with numerous disease processes, and is critical to carcinogenesis particularly through facilitating invasion and cancer cell spread [23]. The gap junction protein connexin43 (Cx43) is understood to be both tumor suppressive and oncogenic, depending on the stage/phase of cancer [24]. Recent studies have shown that increased Cx43 levels correlate with TMZ resistance in GBM cells [25–27]. In addition, brain metastatic cells utilize Cx43 to communicate with normal astrocytes to support tumor growth, invasion, and chemoresistance [28]. Importantly, Cx43 has also been associated with anti-proliferative effects in glioma and reduced levels of Cx43 protein was reported in high-grade gliomas, which highlights a complex dual role for Cx43 in GBM [29, 30].

Cx43 is a four-transmembrane protein that oligomerizes to form connexon hemichannels at the trans-Golgi network before trafficking to the plasma membrane through vesicular transport along microtubules. Once at the cell surface, connexons on apposing cells couple to form gap junction channels that cluster together and allow the passage of small molecules (< 1 kDa), including ions and several second messengers [31]. Cx43 is associated with regulation of a variety of cellular functions, which it effects through channel -dependent and -independent mechanisms; including regulation of cell proliferation, migration/invasion, and apoptosis [24]. Therefore, detailed analysis of the subcellular localization of Cx43, rather than its expression alone, is critical in understanding the relationship between Cx43 and GBM progression.

Low levels of Cx43 expression and associated gap junctions have been observed in GSCs in previous studies [32, 33]. Given the significant non-junctional roles of Cx43 associated with cancer progression, we sought to isolate the function of Cx43 in these GSCs that reportedly have few gap junctions. Using super-resolution Stochastic Optical Reconstruction Microscopy (STORM), we primarily observed intracellular Cx43 decorating microtubules in GSCs, demonstrating for the first time such clustering in situ. The Cx43 protein harbors multiple protein-protein interaction motifs within its cytosolic carboxy-terminus (CT), including a tubulin binding domain [29, 34]. To test the role of Cx43 interacting with microtubules in GSCs, we utilized a Cx43 mimetic peptide named JM2 (juxtamembrane 2) composed of the Cx43 CT amino acids 231–245 encompassing the microtubule binding sequence, and an antennapedia cell penetration domain that promotes cellular uptake [35, 36]. Our data show that JM2 efficiently disrupts interaction between Cx43 and microtubules, and significantly decreases TMZ resistant GSC survival in vitro, and GSC-derived and GBM patient-derived xenograft tumor growth in vivo. Together, these results identify a novel tumorigenic channel-independent role of Cx43 in GSCs through direct interaction with microtubules. JM2 could represent a novel therapeutic peptide specifically modulating Cx43 tumorigenic function to eradicate TMZ-resistant GSCs and improve GBM treatment through delaying tumor recurrence.

## Material and Methods

### Cell culture

Human glioblastoma stem-like cell (GSC) lines LN229/GSC (parental LN229 cells obtained from ATCC), and primary GSCs VTC-001, VTC-034, and VTC-037 were previously isolated and established [27, 37, 38]. GSCs were maintained in serum-free stem cell medium comprising Dulbeccoʼs Modified Eagle Medium (DMEM) with high glucose and L-glutamine (Genesee Scientific), containing Gibco B-27 supplement (Thermo Fisher), fibroblast growth factor-2 (20 ng/ml; PeproTech), epidermal growth factor (20 ng/ml; PeproTech), penicillin / streptomycin (Genesee Scientific), and MycoZAP Plus-PR (Lonza). GSCs were passaged using TrypLE Express (Gibco). Patient-derived xenograft (PDX) GBM22 cells were derived from primary brain tumor tissue, maintained by serial passage in the flank of athymic nude mice as previously described [39], and isolated for in vitro and in vivo experiments (see Animals section below). Following isolation, GBM22 cells were maintained as gliospheres in serum-free medium of DMEM/nutrient mixture F-12 (DMEM/F-12 without phenol red; Thermo Fisher) with L-glutamine, supplemented with sodium pyruvate (Thermo Fisher), Gibco B-27 supplement without vitamin A (Thermo Fisher), 10 ng/ml fibroblast growth factor (Thermo Fisher), 10 ng/ml epidermal growth factor (Thermo Fisher), amphotericin (Fisher), and gentamycin (Fisher). GBM22 cells were dissociated with Accutase (Sigma-Aldrich). To induce GSC differentiation, 10% Fetal Bovine Serum (FBS; Gibco) was directly added to the serum-free medium, or medium was changed to DMEM with high glucose and L-glutamine, penicillin / streptomycin, MycoZAP Plus-PR, and 10% FBS. Normal human astrocytes (NHA) were purchased from Lonza, and cultured in AGM™ Astrocytes Growth Medium BulletKit™ (Lonza). NHA were passaged using Trypsin 0.25% (Quality Biological) after one wash with Phosphate Buffer Saline (PBS; Genesee Scientific) without Ca^++^ and Mg^++^. MycoZAP Plus-PR was employed as antibiotic in all cultures to prevent mycoplasma contamination.

### Peptide and temozolomide treatment

JM2 peptide includes the Cx43 C-terminal amino acids 231–245 that encompass the tubulin binding sequence (VFFKGVKDRVKGRSD). A control peptide, JM2-scrambled, was generated by random re-assembly of the same residues (GSDVFKRKFVDRKVG). Both peptides contain a 16-amino acid antennapedia internalization sequence (RQPKIWFPNRRKPWKK) and are N-terminally biotinylated. Lyophilized peptides were obtained from Peptron, Inc. (South Korea) with purity > 95%, reconstituted in water or in PBS at a concentration of 10 mM, and aliquots were stored at −80 °C freezer. Reconstituted peptides were added to the cell culture medium at different concentrations for times indicated. Temozolomide (TMZ; Selleck Chemicals) was reconstituted in dimethyl sulfoxide (DMSO; Sigma-Aldrich) at a concentration of 50 mM and aliquots were stored at −80 °C freezer. TMZ was directly added to the cell culture medium at different concentrations for times indicated, and DMSO used as vehicle control.

### Gliosphere formation assays

GSCs and PDX GBM22 cells were plated as single cell suspension in low-attachment 96-well plates (GSCs: 100–500 cells per well; GBM22 cells: 500–2000 cells per well), using methods similar to those reported previously [27, 37]. Cells were then treated with peptides every other day. 2 weeks later, gliospheres were observed using phase contrast microscopy on a Revolve microscope (Echo Laboratories). The number of gliospheres was then determined, while the gliosphere size was assessed by diameter measurement. To evaluate stem cell frequency in GSCs, gliosphere formation assay was performed with limiting cell dilutions with cells plated at varying densities in low-attachment 96-well plates. Cells were treated with peptides every other day, and after 2 weeks, gliosphere-positive wells were observed and scored using phase contrast microscopy on a Revolve microscope. The stem cell frequency was analyzed using the online data analysis program ELDA (Extreme Limiting Dilution Analysis) [40].

### MTS viability and caspase 3/7 activity assays

Cells were plated in 96-well plates (2000–5000 cells per well) and treated the following day with TMZ (10, 50, or 100 μM), antennapedia, JM2-scrambled, or JM2 (10, 50, or 100 μM). After 4 days, cell viability was monitored following addition of MTS reagent (Promega) according to manufacturer’s instructions. The absorbance at 490 nm was measured using a SpectraMax i3 or iD3 microplate reader (Molecular Devices). For the caspase 3/7 activity assay, cells were plated in 96-well plates (2000 cells per well) and treated with TMZ or peptides as described above. The activity of caspase 3/7 was measured after 48 h using the Caspase-Glo® 3/7 assay (Promega) following the manufacturer’s instructions. Fold change of caspase 3/7 activity was defined as the ratio of caspase 3/7 luminescence in treated cells compared to controls.

### Western blotting

Cells were lysed in RIPA buffer (0.1% sodium dodecyl sulphate, 50 mM Tris pH 7.4, 150 mM NaCl, 1 mM EDTA, 1% Triton X-100, 1% deoxycholic acid, 200 μM Na_3_VO_4_, and 1 mM NaF) supplemented with Halt^TM^ Protease and Phosphatase Inhibitor Cocktail (Thermo Fisher). Lysates were sonicated prior to centrifugation at 4 °C for 20 min at 10,000 x *g*. Protein concentration was quantified using the DC protein assay (Bio-Rad Laboratories) and a standard curve obtained with increasing concentrations of Bovine Serum Albumin (BSA; Fisher Scientific). Protein samples were normalized to 20 μg total protein per lane and resolved by SDS-PAGE electrophoresis. 4X Bolt LDS sample buffer supplemented with 400 mM DTT (final concentration 1X Bolt LDS with 100 mM DTT) was added to samples before heating at 70 °C for 10 min. SDS-PAGE was run using NuPAGE Bis-Tris 4%–12% gradient gels (Thermo Fisher) with MES or MOPS running buffer according to manufacturer’s instructions. Spectra™ Multicolor Protein Ladder (Thermo Fisher) or Precision Plus Protein™ Kaleidoscope™ Prestained Protein Standards (Bio-Rad Laboratories) were included as protein ladders on the same gels. Proteins were transferred to a PVDF membrane using the Bio-Rad Turbo Transblot System and transfer kit (Bio-Rad Laboratories) and fixed in methanol prior to blocking in 5% non-fat milk in TNT buffer (50 mM Tris pH 8.0, 150 mM NaCl, 0.1% Tween 20) for 2 h at room temperature. Primary antibody labeling was performed overnight at 4 °C using the following primary antibodies diluted in TNT buffer containing 3% BSA; rabbit anti-Cx43 (MilliporeSigma; C6219), rabbit anti-CD133 (Abcam; ab19898), rabbit anti-Olig2 (MilliporeSigma; AB9610), rabbit anti-SOX2 (Cell Signaling Technology; 3579), rabbit anti-Notch1 (Cell Signaling Technology; 3608 and 4380), mouse anti-α-tubulin (MilliporeSigma; T6199), rabbit anti-β-tubulin (Abcam; ab151318), rabbit anti-GAPDH (Santa Cruz Biotechnology; sc-25778, and Cell Signaling Technology; 2118), and mouse anti-GAPDH (Proteintech; 60004-1-Ig). Membranes were washed 3 × 10 min in TNT buffer prior to secondary antibody labeling for 1 h at room temperature with goat secondary antibodies against mouse or rabbit IgG conjugated to HRP (MilliporeSigma; DC02L and SAB3700878). Biotin-tagged peptides were labeled with Pierce™ High Sensitivity NeutrAvidin™-HRP (Thermo Fisher; 31030) directly after blocking. After 3 × 10 min washes in TNT buffer, SuperSignal™ West Pico PLUS Chemiluminescent Substrate or SuperSignal™ West Femto Maximum Sensitivity Substrate (Thermo Fisher) were used according to manufacturer’s instructions prior to imaging. Membranes were imaged on a ChemiDoc™ imaging system, and analyzed with Image Lab software (Bio-Rad Laboratories). When necessary, membranes were re-blotted using Re-Blot Plus Strong Solution (MilliporeSigma) for 1 h before blocking and immunoblotting with different primary antibodies.

### Solubility assay

Cells were lysed in 1% Triton X-100 buffer (50 mM Tris pH 7.4, 1% Triton X-100, 2 mM EDTA, 2 mM EGTA, 250 mM NaCl, 1 mM NaF, 0.1 mM Na_3_VO_4_; supplemented with Halt^TM^ Protease and Phosphatase Inhibitor Cocktail, Thermo Fisher), and rotated for 1 h at 4 °C. At this point, 10% of the lysate was removed and 4X LDS NuPAGE sample buffer (Thermo Fisher) supplemented with 400 mM DTT was added prior to sonication and centrifugation at 10,000 x *g* for 20 min to obtain the ‘total’ fraction. The remaining lysate was fractionated by centrifugation for 30 min at 15,000 x *g* in pre-weighed microcentrifuge tubes. The supernatant was removed and combined with 4X LDS NuPAGE sample buffer prior to sonication and centrifugation at 10,000 x *g* for 20 min to obtain the ‘soluble’ fraction. Pellets were weighed and suspended in 1X LDS NuPAGE sample buffer to a final concentration of 30 mg/ml. Pellets were solubilized with disruption by several passages through an insulin syringe and sonication prior to centrifugation at 10,000 x *g* for 20 min. Protein samples were denatured by heating at 70 °C for 10 min and underwent SDS-PAGE with NuPAGE Bis-Tris 4–12% gradient gels and MES running buffer (Thermo Fisher). SeeBlue™ Pre-stained Protein Standard (Thermo Fisher) was included as protein ladder on the same gel. Proteins were transferred to LF-PVDF membranes (Bio-Rad Laboratories), with membranes methanol-fixed and air-dried post-transfer. Following methanol reactivation, membranes were blocked in 5% nonfat milk (Carnation) in TNT buffer (50 mM Tris pH 8.0, 150 mM NaCl, 0.1% Tween 20) for 1 h at room temperature. Primary antibody incubation was conducted overnight at 4 °C using rabbit anti-Cx43 (1:5,000; MilliporeSigma; C6219) and mouse anti-α-tubulin (1:5,000; MilliporeSigma; T6199). Membranes were rinsed twice and washed 3 × 10 min in TNT buffer. Secondary antibody labeling was conducted for 1 h at room temperature using antibodies conjugated to Alexa Fluor 555 and Alexa Fluor 647 (Thermo Fisher). Following the secondary antibody incubation, membranes were washed in TNT buffer as previously described. Prior to imaging, membranes were fixed in methanol and air dried. Images were acquired using a Chemidoc MP imaging system (Bio-Rad Laboratories). Western blot quantification was conducted using Bio-Rad Image Lab software.

### Co-immunoprecipitation

Prior to lysis, cross-linking was completed by incubating cells in DTBP (dimethyl 3,3’-dithiobispropionimidate; 2 mM in PBS) for 30 min at 37 °C followed by a glycine quench (100 mM glycine in PBS) for 15 min at room temperature. Cross-linked samples were lysed in RIPA buffer supplemented with Halt^TM^ Protease and Phosphatase Inhibitor Cocktail as described above, but without sonication. Co-immunoprecipitation (co-IP) assays were also performed without cross-linking; non-cross-linked samples were lysed using a co-IP lysis buffer (0.5% Triton X-100, 50 mM Tris HCl, 150 mM NaCl, 1 mM EDTA, 1 mM EGTA, 1 mM DTT, 0.1 mM Na_3_VO_4_, and 1 mM NaF) supplemented with Halt^TM^ Protease and Phosphatase Inhibitor Cocktail, without sonication. Protein concentration was quantified using the DC protein assay and a standard curve obtained with increasing concentrations of BSA, and 500 μg of total protein was used per reaction for co-IP. Inputs were removed prior to co-IP and denatured in Bolt LDS sample buffer as described above. Remaining protein lysates were incubated with Protein G Sepharose^TM^ 4 Fast Flow beads (GE Healthcare) for pre-clearance for 30 min at 4 °C. Samples were incubated with 2 μg of mouse anti-α-tubulin (MilliporeSigma, T6199) for cross-linked samples or mouse anti-Cx43 antibody (EMD Millipore; MAB3067) for non-cross-linked samples for 90 min at 4 °C. Mouse-IgG (R&D Systems™; MAB002), mouse antibodies against HA.11 tag (Biolegend; 901513), or V5 tag (Cell Signaling Technology; 80076) were used as isotype negative controls. Samples were then incubated with Protein G Sepharose^TM^ 4 Fast Flow beads for 60 min at 4 °C. Protein complexes were washed four times with RIPA or co-IP lysis buffers, then eluted and denatured in 2X Bolt LDS buffer supplemented with 200 mM DTT. SDS-PAGE and western blotting occurred as described above using rabbit anti-Cx43 (MilliporeSigma; C6219) or rabbit anti-β-tubulin (Abcam; ab151318) primary antibodies.

### Immunofluorescence

Cells were washed once with warm PBS before fixing in 4% paraformaldehyde for 20 min at room temperature or ice-cold methanol for 5 min on ice, washed twice, and held in PBS at 4°C until immunostaining was conducted. Cells were permeabilized and blocked in 5% normal goat or donkey serum (Fisher Scientific) and 0.1% Triton X-100 in PBS for 2 h at room temperature. Primary antibodies rabbit anti-Cx43 (MilliporeSigma; C6219), mouse anti-pan Cadherin (MilliporeSigma; C1821), or mouse anti-α-tubulin (MilliporeSigma; T6199) were diluted in blocking buffer and labeling was performed overnight at 4 °C. Cells were washed six times with PBS (2 × quick, 2 × 10 min, 2 × 5 min) prior to incubating with goat anti-mouse or -rabbit IgG secondary antibodies (Thermo Fisher) conjugated to Alexa Fluor 488 (A-11029; A-11034), Alexa Fluor 555 (A-21424; A-21429), or Alexa Fluor 647 (A-21236; A-21245) for confocal; or donkey anti-mouse or -rabbit IgG secondary antibodies conjugated to CF568 (Biotium; 20105 and 20098) or Alexa Fluor 647 (Jackson Immunoresearch; 715-605-151 and 711-605-152) for stochastic optical reconstruction microscopy (STORM; see section below), for 1 h at room temperature. DAPI (Thermo Fisher) was included with secondary antibodies to counterstain nuclei for confocal only and was omitted from labeling for STORM. Cell membranes were labeled using Alexa Fluor 488- or Alexa Fluor 647-conjugated wheat germ agglutinin (WGA; Thermo Fisher; W11261 and W32466). Slides were washed 6 times as before and mounted using Prolong Gold Antifade (Thermo Fisher) for confocal microscopy, or maintained in PBS at 4°C prior to STORM imaging. To detect biotin-tagged peptides, fixed cells were incubated after the first set of washes with streptavidin conjugated to Alexa Fluor 647 (Thermo Fisher) diluted in high-salt buffer (0.5 M NaCl, 10 mM Hepes) for 30 min at room temperature. Cells were then washed four times with high-salt buffer (2 × quick, 2 × 10 min), and twice with PBS (2 × 5 min) before proceeding with secondary antibodies and washes as described above. For confocal microscopy, images were acquired using an Opterra inverted confocal microscope (Bruker) and 60x or 100x objective (Nikon) equipped with an EMCCD Photometrics Evolve Delta detector. Acquisitions were performed as Z-series with 0.5 µm step sizes, and analyses undertaken with imageJ software (NIH). Investigators were blinded for all image acquisition experiments and analyses.

### Super-resolution (STORM) localization and analysis

Stochastic optical reconstruction microscopy (STORM) acquisition was conducted with a Vutara 350 microscope (Bruker). Immunolabeled cells were imaged in 50 mM Tris-HCl, 10 mM NaCl, 10% (wt/vol) glucose buffer containing 20 mM mercaptoethylamine, 1% (vol/vol) 2-mercaptoethanol, 168 active units/ml of glucose oxidase, and 1404 active units/ml catalase. 2500 frames were acquired for each probe and 3D images were reconstructed in Vutara SRX software. Coordinates of localized molecules were used to calculate pair correlation functions in the Vutara SRX software. To quantify the spatial relationship between target proteins localized by STORM, we utilized cross-pair correlation analysis. Resulting correlation curves identify peaks of actual association between molecules in 5 nm increments. A pair correlation less than an amplitude of 1 indicates negative co-localization between two proteins and higher values indicating a positive correlation. Investigators were blinded for STORM acquisition and analyses.

### Cellular thermal shift assay (CETSA)

For an initial determination of the melting profile of α- and β- tubulin, fresh lysates of GSCs prepared in non-denaturing buffer were dispensed into 96-well PCR plate in stem cell medium (approximately 10,000 cells/well/50 µl), and then subjected to temperature gradient (37–65 °C) for 20 min. Subsequently, centrifugation was performed at 14,000 rpm to sediment the unstable protein content. Supernatant was collected and SDS-PAGE gel was run, and immunodetection was performed to detect α- and β- tubulin using specific primary antibodies described above. A rabbit antibody against β-actin (Cell Signaling Technology; 4970) was used as negative control. Band intensities were quantified on LI-COR C-DiGit Blot Scanner, and subsequently T_agg_(50) and T_agg_(75) values were calculated for α- and β- tubulin. In a subsequent run, fresh lysates of GSCs were treated at various doses with 3-fold dilutions (222.2, 74.0, 24.7, 8.2 and 2.7 nM) of peptides JM2 and JM2-scrambled, together with DMSO as control, for 1 h. Samples were then subjected to heat challenge at 57 °C for 20 min, and unstable protein was removed by centrifugation step. Following an immunoblotting step, band intensities of the stable tubulin was quantified, and normalized to DMSO control. EC50 values of engagement for both peptide with α- and β- tubulin were subsequently calculated.

### Reverse transcriptase quantitative PCR

RNA was extracted using the PureLink^TM^ RNA mini extraction kit (Thermo Fisher) and on-column DNA digestion was completed using PureLink^TM^ DNase according to the manufacturer’s instructions. RNA was reverse transcribed to generate cDNA using the iScript^TM^ Reverse Transcription SuperMix for RT-qPCR kit (Bio-Rad Laboratories). Real-time PCR was performed using the SYBR select Master Mix for CFX (Thermo Fisher) in hard-shell 96-well PCR plates (Bio-Rad Laboratories) on a CFX Connect Real Time System (Bio-Rad Laboratories). Primer sequences used were: hNOTCH1_Fwd: 5’-CGCACAAGGTGTCTTCCAG-3’; hNOTCH1_Rev: 5’-CGGCGTGTGAGTTGATGA-3’; hHES1_Fwd: 5’-GGCTGGAGAGGCGGCTAA-3’; hHES1_Rev: 5’-GAGAGGTGGGTTGGGGAGTT-3’; hHEY1_Fwd: 5’-ACGAGACCGGATCAATAACA-3’; hHEY1_Rev: 5’-ATCCCAAACTCCGATAGTCC-3’.

### Animals

Animal studies were approved by the Institutional Animal Care and Use Committee (IACUC) of Virginia Tech. Female athymic nude mice (Envigo) aged 6-8 weeks were used for the maintenance and propagation of PDX GBM22 cells. PDX GBM22 tumors were maintained by serial passage in nude mouse flanks as described previously [39]. Briefly, tumors were harvested 14–18 days post-injection and glioblastoma cells were mechanically and enzymatically dissociated. Cells were passed through a 40 µm filter and maintained as gliospheres in DMEM/F-12 without phenol red and with L-glutamine (Thermo Fisher), supplemented with 10 ng/ml fibroblast growth factor (Thermo Fisher), 10 ng/ml epidermal growth factor (Thermo Fisher), Gibco B-27 Supplement without vitamin A (Thermo Fisher), amphotericin (Fisher), and gentamycin (Fisher). The medium was changed daily for 2 days. GBM22-derived gliospheres were maintained in vitro for 5–7 days before dissociation with Accutase (Sigma-Aldrich), and cell counting for gliosphere formation assays (see section above) or tumor formation in mouse flank in vivo. For the latter, GBM22 cells were diluted in sterile PBS and between 2 and 2.5 × 10^6^ cells were subcutaneously injected in the flanks of 6–8-week old female athymic nude mice (Envigo). After 2 days, tumors were measured and mice were randomly separated into three groups (7 mice per group), and vehicle/control (PBS), JM2-scrambled or JM2 were administered at 300 μM intratumorally every day for 14 days. Tumors were then measured by blinded investigators and harvested for analysis (see section below). Tumor volume was calculated using the formula (length × width^2^)/2. For the LN229/GSC tumor model (Supplementary Fig. 3), LN229/GSCs (1 × 10^6^) were mixed with Corning Matrigel® matrix growth factor reduced and subcutaneously injected into flanks of 8-week old male BALB/c nude mice (Charles River Laboratories) as previously described [27, 38]. After 7 days, tumors were measured and mice were randomly separated into three groups; vehicle/control (PBS), JM2-scrambled, and JM2 (3 mice per group). At day 9, vehicle or peptides were administered at 300 μM intratumorally every other day for 24 days. Tumor sizes were measured by electronic calipers at different times by blinded investigators, and tumor volume was calculated using the formula (length × width^2^)/2.

### Tissue collection, sectioning, staining, and image acquisition

Tumors were harvested, snap-frozen in Tissue-Tek® O.C.T. compound (Sakura), and stored at −80 °C until sectioning. 10 μM sections were cut using a Leica CM1850 UV cryostat, placed onto Superfrost Plus^TM^ slides (Fisher Scientific) and fixed in −20 °C acetone for 5 minutes before air drying. Slides were stored at −80 °C prior to labeling. Tumors sections were then rehydrated in PBS for 5 min, permeabilized in 0.1% Triton X-100 in PBS for 1 h at room temperature, and washed four times with PBS (2 x quick, 2 × 5 min) prior to incubating with streptavidin conjugated to Alexa Fluor 647 (Thermo Fisher) diluted in high-salt buffer (0.5 M NaCl, 10 mM Hepes) for 30 min at room temperature. Tumor sections were then washed four times with high-salt buffer (2 x quick, 2 × 10 min), and twice with PBS (2 × 5 min) before using One-step TUNEL In Situ Apoptosis kit (Green Elab Fluor® 488; Elabscience), according to manufacturer’s instructions. Tumor sections were then washed in PBS (3 × 5 min), incubated with DAPI (Thermo Fisher) in PBS for 5 min, and washed again with PBS (4 × 5 min) before the slides were mounted using Prolong Gold Antifade (Thermo Fisher). GBM22-derived tumor sections were analyzed using fluorescence confocal microscopy on a Nikon NIE upright confocal microscope, while LN229/GSC-derived tumor sections were observed using LED illumination microscopy on a Revolve microscope. For hematoxylin and eosin (H&E) staining, tumor sections were stained using a Histo-Tek® SL Slide Stainer (Sakura), and observed using phase contrast microscopy on a Revolve microscope. Investigators were blinded for all image acquisition experiments.

### Statistical analyses

All quantification was performed on experiments repeated at least three times. Statistical analysis was conducted with GraphPad Prism (GraphPad Software). Data were analyzed for significance using Student’s t test, one-way ANOVA with Tukey’s multiple comparisons test, and presented as mean ± SD. A value of *p* < 0.05 was considered statistically significant. Extreme Limiting Dilution Analysis (ELDA) was performed using the ELDA software tool (bioinf.wehi.edu.au/software/elda) to estimate GSC frequency and compare conditions statistically. The software models the data using a generalized linear model with a complementary log-log link function, assuming a single-hit Poisson process for limiting dilution. Statistical significance between experimental groups was determined using chi-square (χ²) tests within the ELDA framework. Confidence intervals (CIs) for GSC frequency were reported at 95%. A within-between interaction repeated measure ANOVA was performed to evaluate differences in the rate of tumor growth among interventions for the LN229/GSC tumor model (Supplementary Fig. 3). Specifically, value was the dependent variable and group, time, and a group by time interaction were the independent variables. A random effect was included for each mouse to handle the repeated measures. Data are presented as mean ± SEM.

## Results

### Cx43 displays increased cytoplasmic interaction with microtubules in glioblastoma stem-like cells

**Fig. 1:**
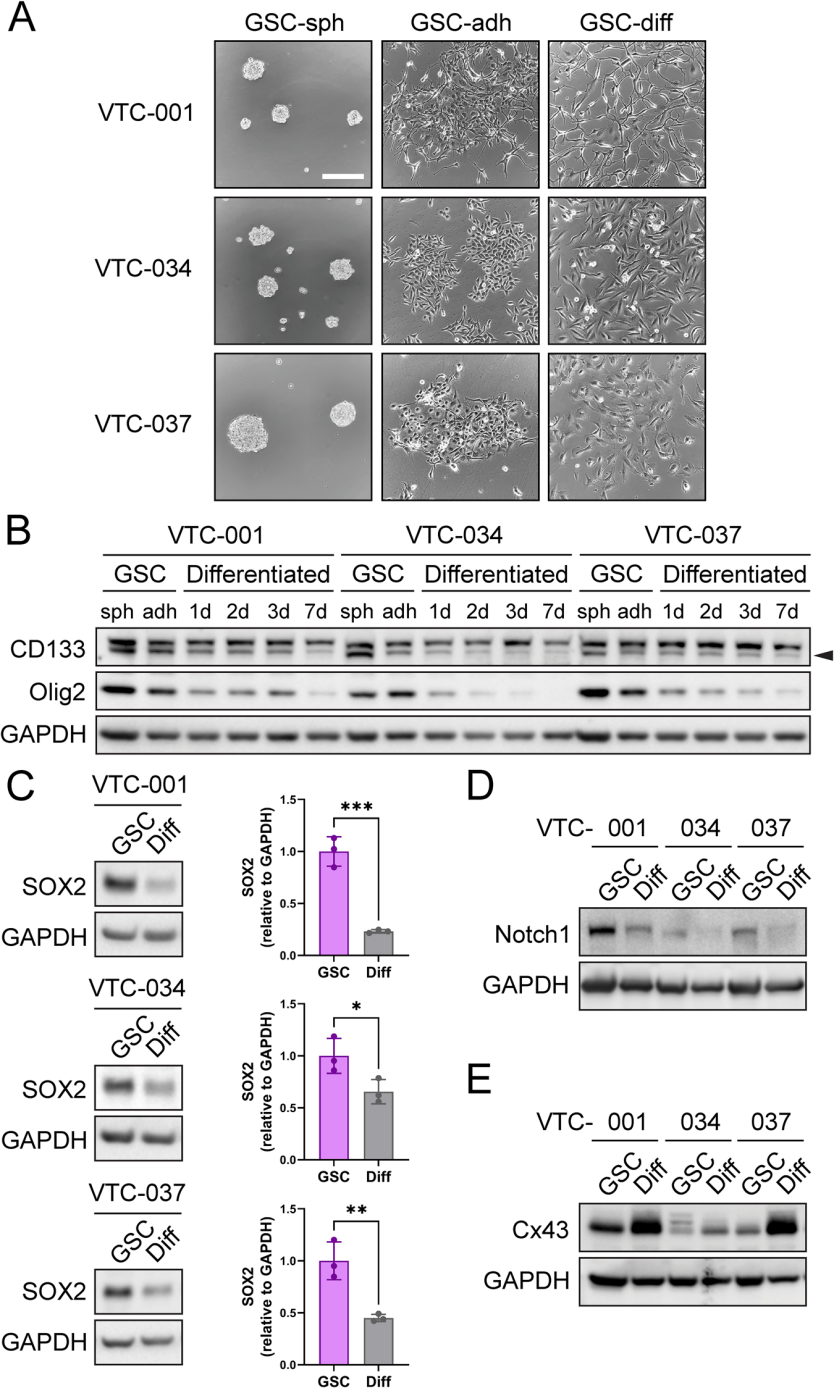
Stemness marker and Cx43 expression during GSC differentiation. **A** VTC-001, VTC-034, and VTC-037 GSCs were cultured in stem cell medium as gliospheres (GSC-sph) or as adherent cells (GSC-adh), or differentiated for 1 to 3 days in medium containing 10% FBS (GSC-diff), and observed by phase-contrast microscopy. Scale bar: 200 μm. **B** VTC-001, VTC-034, and VTC-037 cultured as GSC-derived gliospheres (sph), adherent GSCs (adh), or differentiated cells after addition of 10% FBS for 1, 2, 3, and 7 days, were lysed and analyzed by immunoblotting using antibodies against CD133, Olig2, and GAPDH as loading control. **C** VTC-001, VTC-034, and VTC-037 GSCs were differentiated (Diff) or not for 24 h, and cell lysates were analyzed by immunoblotting for SOX2, and GAPDH as loading control (quantification shown on the right; *n* = 3). A two-tailed unpaired Student’s *t*-test was used; *p ≤ 0.05, **p ≤ 0.01, ***p ≤ 0.001. Data are represented as mean ± SD. **D** VTC-001, VTC-034, and VTC-037 GSCs were differentiated (Diff) or not for 24 h, and cell lysates were analyzed by immunoblotting for Notch1, and GAPDH as loading control. **E** VTC-001, VTC-034, and VTC-037 GSCs were differentiated (Diff) or not for 24 h, and cell lysates were analyzed by immunoblotting for Cx43, and GAPDH as loading control.

Primary human glioblastoma stem-like cells (GSCs) VTC-001, VTC-034, and VTC-037 previously isolated from freshly resected GBM tumors [27, 37] were cultured in stem cell medium as GSC-derived gliospheres or adherent GSCs, or differentiated by addition of 10% FBS (Fig. 1A). Stemness was confirmed in gliospheres and adherent GSCs by western blotting, as observed by expression of GSC markers CD133 and Olig2 (Fig. 1B), SOX2 (Fig. 1C), and Notch1 (Fig. 1D). FBS-induced differentiation caused a decrease in CD133 and Olig2 expression after 24 h, which was further accentuated after 7 days (Fig. 1B). Similarly, SOX2 and Notch1 expression both significantly decrease 24 h after addition of FBS (Fig. 1C, D). Interestingly, we observed an increase in Cx43 expression at the protein level in differentiated VTC-001, VTC-034, and VTC-037 compared to their GSC counterparts (Fig. 1E).

**Fig. 2:**
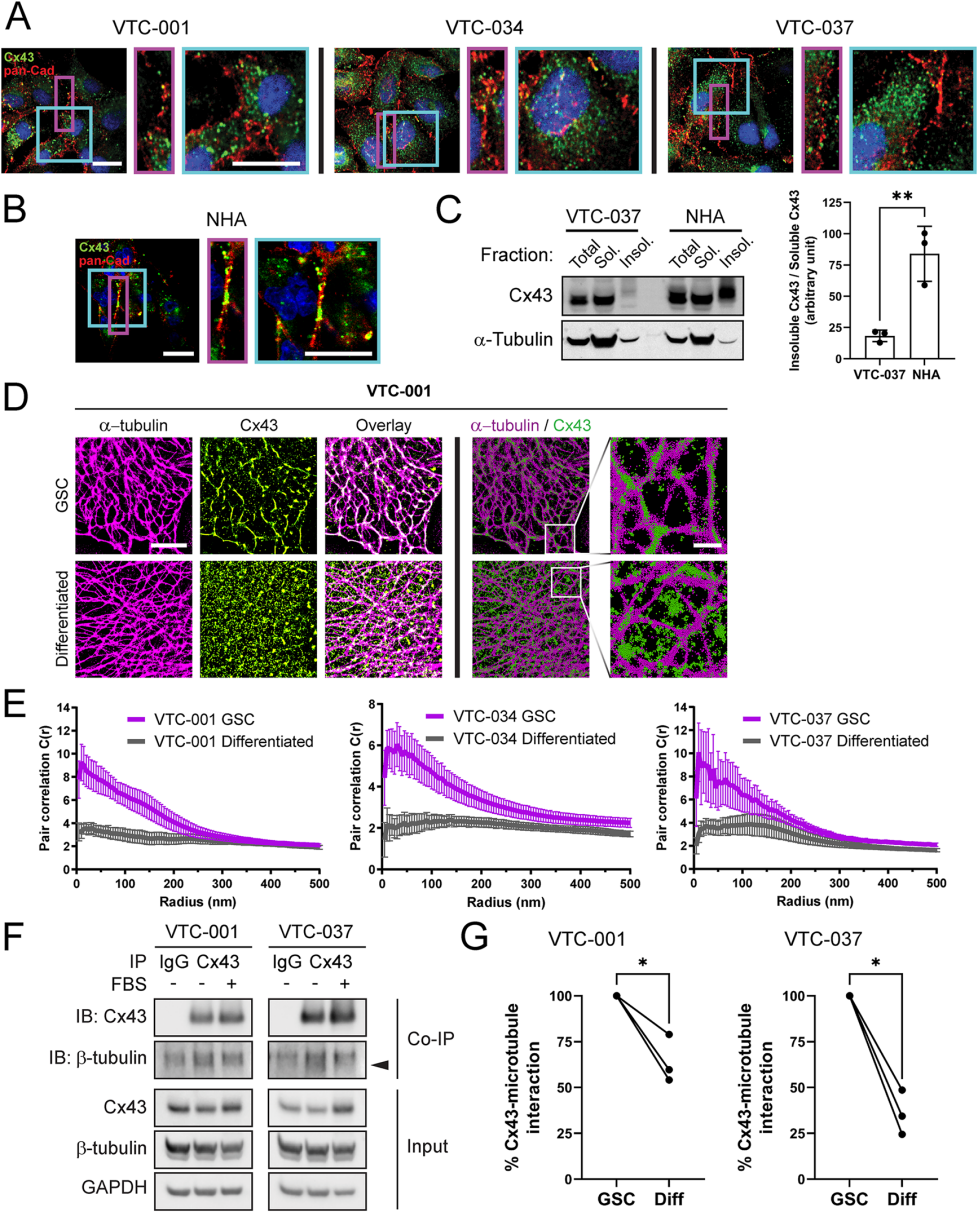
Increased Cx43 interaction with microtubules in GSCs. VTC-001, VTC-034, and VTC-037 GSCs (**A**), or normal human astrocytes (NHA; **B**) in culture were fixed and immunostained using antibodies against Cx43 (green), and pan-Cadherin (pan-Cad – red) to stain cell borders. Confocal immunofluorescence was used to observe Cx43 subcellular localization in the cytoplasm and at the cell borders. DAPI was used to stain nuclei. Scale bar: 20 μm. **C** VTC-037 GSC protein lysates were subjected to fractionation using a Triton X-100 solubility assay, and analyzed by immunoblotting for total, soluble (non-junctional), and insoluble (junctional) Cx43. Quantification of insoluble (junctional) Cx43 band intensity relative to soluble (non-junctional) shown on the right (*n* = 3). A two-tailed unpaired Student’s *t*-test was used; **p ≤ 0.01. Data are represented as mean ± SD. **D** Stochastic optical reconstruction microscopy (STORM) derived localizations of Cx43 (green) and α-tubulin (magenta) in VTC-001 GSCs or differentiated by addition of 10% FBS for 24 h. Left 6 panels: point-splatting visualization of STORM localizations to better identify complexing (white; scale bar: 5 μm). Right 4 panels: point-clouds of 50 nm spheres representing individual localizations, including zoomed-in regions (scale bar: 1 μm). **E** Cross-pair correlation functions for Cx43/α-tubulin complexing in VTC-001, VTC-034, and VTC-037 GSCs versus differentiated cell populations. (*n* = 10). **F** VTC-001 and VTC-037 GSCs were differentiated or not, and cell lysates were subjected to co-immunoprecipitation using Cx43 antibody or IgG for negative control, and/or immunoblotted using antibodies against Cx43, β-tubulin, and GAPDH for loading control. Black arrow indicates β-tubulin band. **G** Densitometry quantification of β-tubulin levels co-immunoprecipitating with Cx43 in differentiated cells relative to GSC counterparts (*n* = 3; decreased: 40.3%, 21.1%, and 45.9% in differentiated VTC-001; decreased: 51.4%, 65.6%, and 75.5% in differentiated VTC-037). A two-tailed unpaired Student’s *t*-test was used; *p ≤ 0.05.

Although Cx43 expression was lower in GSCs, confocal immunofluorescence microscopy revealed Cx43 primarily enriched within the cytoplasm of GSCs (Fig. 2A), which is in contrast to subcellular localization of Cx43-associated gap junctions primarily observed at the plasma membranes in normal human astrocytes (NHA; Fig. 2B). To complement these studies biochemically, we implemented a Triton X-100 solubility assay to measure insoluble (gap junction) versus soluble (non-junctional) species of Cx43. Just as we saw with microscopy, we found a significant decrease in Cx43 in the insoluble (gap junction) fraction in VTC-037 GSCs compared to NHA, where insoluble gap-junctional Cx43 was highly enriched (Fig. 2C). We then utilized Stochastic Optical Reconstruction Microscopy (STORM) to further investigate the subcellular localization of Cx43 (green) in GSCs and found clear colocalization with the microtubule cytoskeleton (magenta) in VTC-001, VTC-034, and VTC-037 GSCs compared to differentiated GSCs (Fig. 2D, Supplementary Fig. 1A and B). Differentiating GSCs in medium containing 10% FBS resulted in a significant redistribution of Cx43 away from this direct interaction with microtubules (Fig. 2D, Supplementary Fig. 1A and B), quantified by pair correlation analysis (Fig. 2E). Values greater than 1 at 5 nm indicate positive complexing, and quantification of these values and subsequent decay are used to measure changes in protein-protein interaction. These results were confirmed biochemically by co-immunoprecipitation (co-IP), where again, decreased interaction between tubulin and Cx43 was observed following addition of 10% FBS in VTC-001 and VTC-037 GSCs (Fig. 2F; lower band indicated by black arrow). Quantification of three independent co-IP experiments confirms a significant decrease in Cx43 interaction with microtubules in GSCs post-differentiation (Fig. 2G).

### Cx43 mimetic peptide JM2 disrupts Cx43 interaction with microtubules in GSCs

**Fig. 3:**
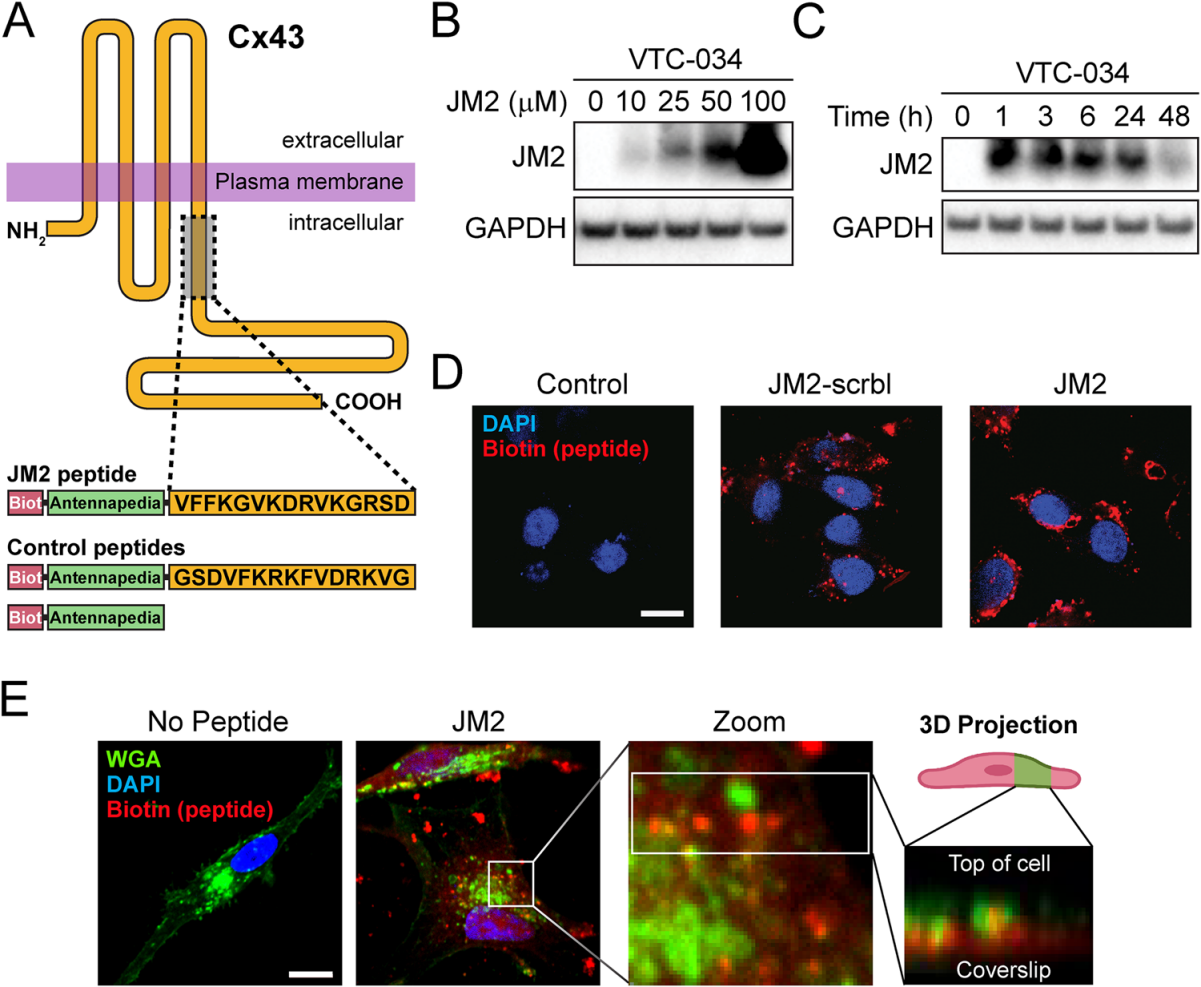
Cx43 mimetic JM2 peptide cell uptake in GSCs. **A** Schematic of the JM2 peptide that encompasses the Cx43 tubulin-binding domain, an antennapedia cell penetration domain, and a biotin tag for tracking. Control peptides include JM2-scrambled and antennapedia. **B** VTC-034 GSCs were treated or not with JM2 at different concentrations for 24 h, lysed and analyzed by blotting using Neutravidin-HRP detection, and immunoblotting for GAPDH as loading control. **C** VTC-034 GSCs were treated with JM2 at 50 μM for different times, lysed and analyzed by blotting using Neutravidin-HRP, and immunoblotting for GAPDH as loading control. **D** VTC-034 GSCs were treated or not with JM2-scrambled (JM2-scrbl) or JM2 at 50 μM for 24 h, and fixed. Biotin-tagged JM2-scrambled and JM2 were observed by confocal fluorescence microscopy using fluorophore-conjugated streptavidin (red), and DAPI was used to stain nuclei. Scale bar: 10 μm. **E** Fluorescence confocal microscopy of VTC-037 GSCs treated or not with JM2 at 50 μM for 24 h before fixing, and probed for Wheat Germ Agglutinin (WGA – green), biotin-tagged JM2 (red), with nuclei identified using DAPI (blue). Zoom and 3D projection on right. Scale bar: 10 μm.

To assess the role of Cx43/microtubule interaction in GSCs, we utilized a Cx43 mimetic peptide, JM2, encompassing the Cx43 tubulin-binding domain, an antennapedia cell penetrating sequence, and a biotin tag for tracking, with biotin-tagged antennapedia and JM2-scrambled as negative controls (Fig. 3A) [35, 36, 41]. Cellular uptake of JM2 at increasing concentrations was confirmed by western blotting in VTC-034 GSCs using HRP-conjugated Neutravidin for detection (Fig. 3B). JM2 cell uptake was apparent after 1 h, and sustained for 24 h before JM2 signal in GSCs decreased at 48 h (Fig. 3C). Peptide uptake in GSCs was assessed by confocal fluorescence microscopy, where JM2 and JM2-scrambled (red) were confirmed to be localized within the cytoplasm of GSCs (Fig. 3D). To further confirm that administered peptides were truly cytoplasmic, we performed Z-stack analyses using WGA (green) to identify post-Golgi cell membranes. 3D-projection of Z-stacks revealed enrichment of JM2 (red) within cells, as identified by localizations subjacent to WGA-labeled cell surfaces and colocalization with intracellular WGA signal (Fig. 3E).

**Fig. 4:**
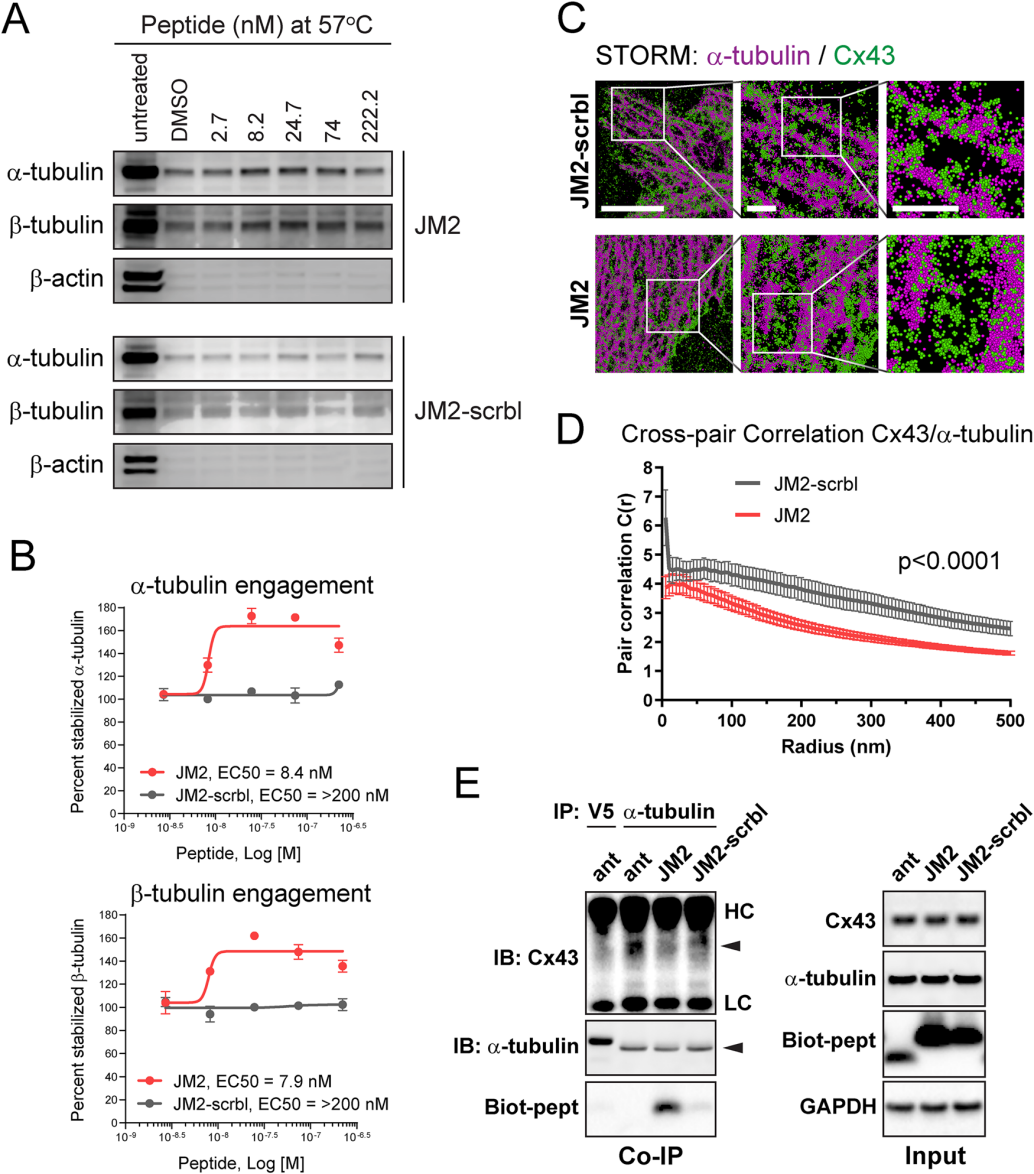
JM2 disrupts Cx43 – microtubule interaction. **A** Cellular thermal shift assay in VTC-037 GSC lysates was used to determine JM2 and JM2-scrambled selective target engagement potency for α-tubulin and β-tubulin. VTC-037 GSC lysates were subjected to different concentration of JM2 or JM2-scrambled (JM2-scrbl) at 57 °C, and peptide affinity was analyzed by western blotting using antibodies against α-tubulin, β-tubulin, and β-actin as negative control. **B** Percentage of stabilized α-tubulin and β-tubulin at 57 °C was represented, and EC50 values for JM2 and JM2-scrambled (JM2-scrbl) were calculated. **C** STORM derived point-cloud localizations of Cx43 (green) and α-tubulin (magenta) in VTC-037 GSCs following treatment with JM2-scrambled (JM2-scrbl) or JM2 at 50 μM for 24 h. Zoomed out panels (left) scale bar: 6 μm. Zoomed in panels (middle) scale bar: 1 μm. Zoomed in panels (right) scale bar: 1 μm. Sphere size: 50 nm. **D** Cross-Pair correlation functions for Cx43/α-tubulin interaction in C (*n* = 10). **E** VTC-037 GSCs were treated or not with antennapedia (ant), JM2, or JM2-scrambled (JM2-scrbl) at 50 μM for 24 h. Following cross-linking, cell lysates were subjected to co-immunoprecipitation using α-tubulin antibody, or V5 antibody for negative control, and immunoblotted using antibodies against Cx43, α-tubulin, and GAPDH for loading control. Neutravidin-HRP was used to detect biotin-tagged peptides. HC heavy chain, LC light chain.

We next performed cellular thermal shift assay (CETSA) to characterize the specificity and affinity of JM2 interaction with α- and β- tubulin, i.e., primary microtubular subunits. In an initial heat gradient, thermal melting profiles of α- and β- tubulin were determined in VTC-037 GSC lysates with T_agg_(50) values of 49.5 °C for α-tubulin, and 49.2 °C for β-tubulin (Supplementary Fig. 2). T_agg_(75) values for α- and β-tubulin were calculated as 57 °C. Subsequent dose-gradients of the peptides JM2 and JM2-scrambled under T_agg_(75) revealed their selective target engagement potency (EC50) for α-tubulin and β-tubulin, while no EC50 was detected for actin, which was used as a negative control (Fig. 4A). EC50 values for JM2 were calculated as 8.4 nM for α-tubulin, and 7.9 nM for β-tubulin, while EC50 values for JM2-scrambled were above 200 nM for both α-tubulin and β-tubulin (Fig. 4B). These data confirmed that JM2 presented higher potency of target engagement with α-tubulin and β-tubulin compared to the JM2-scrambled control peptide. We then employed STORM localization analysis to test whether JM2 disrupts Cx43−microtubule interaction in VTC-037 GSCs, and found that JM2 impaired this interaction in situ (Fig. 4C, D). These results were biochemically confirmed by co-IP experiments, wherein it was determined that JM2 robustly interacted with α-tubulin compared to antennapedia alone or JM2-scrambled peptides, concomitantly with a decrease in Cx43 interaction with α-tubulin (Fig. 4E). Importantly, ‘input’ results indicate JM2 does not affect Cx43 expression in GSCs (Fig. 4E; right panel).

### JM2 decreases GSC survival and self-renewal

**Fig. 5:**
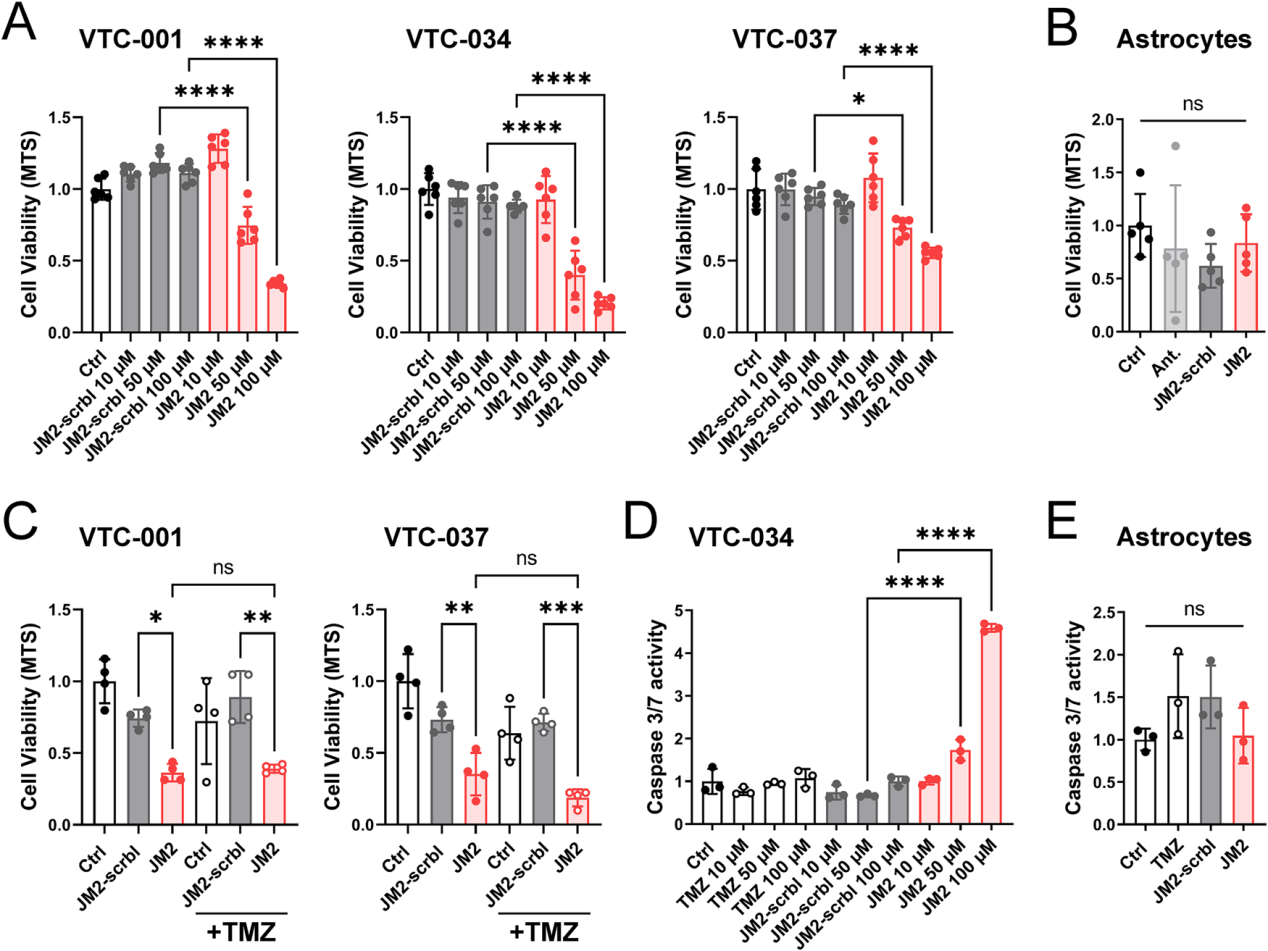
JM2 inhibits GSC survival. **A** VTC-001, VTC-034, and VTC-037 GSCs were cultured as adherent cells in 96-well plates and treated or not with 10, 50 or 100 μM of JM2-scrambled (JM2-scrbl) or JM2 peptides for 4 days before cell viability was assessed using MTS assay. **B** Human astrocytes cultured in 96-well plates were treated or not with 100 μM of antennapedia (ant), JM2-scrambled (JM2-scrbl) or JM2 peptides for 4 days before cell viability was assessed using MTS assay. **C** VTC-001 and VTC-037 GSCs were cultured as adherent cells in 96-well plates and treated or not with temozolomide (TMZ) at 50 μM, with or without JM2-scrambled (JM2-scrbl) or JM2 peptides at 100 μM for 4 days before cell viability was assessed using MTS assay. **D** VTC-034 GSCs were cultured as adherent cells in 96-well plates and treated or not with different concentrations of TMZ, JM2-scrambled (JM2-scrbl), or JM2 for 48 h at 10, 50 or 100 μM before assessing apoptosis using caspase 3/7 assay. **E** Human astrocytes cultured in 96-well plates were treated or not with 100 μM of TMZ, JM2-scrambled (JM2-scrbl) or JM2 for 2 days before assessing apoptosis using caspase 3/7 assay. Statistical analysis was performed with one-way analysis of variance (ANOVA) with Tukey’s multiple comparisons test. *p ≤ 0.05, **p ≤ 0.01, ***p ≤ 0.001, ****p < 0.0001, ns not significant. Data are represented as mean ± SD.

We next determined the effect of JM2 in GSCs and observed that JM2 significantly decreased VTC-001, VTC-034, and VTC-037 GSC survival when cultured as adherent cells in a dose-dependent manner using an MTS assay (Fig. 5A). As Cx43 is primarily expressed in astrocytes in the central nervous system [42], we tested the effect of JM2 in these non-tumor cells. We did not observe a decrease in human astrocyte viability, even at higher JM2 concentrations (Fig. 5B). As we previously reported a relationship between Cx43 and TMZ resistance, we tested the combination of TMZ and JM2 on GSC viability. While VTC-001 and VTC-037 GSCs are resistant to TMZ treatment, as previously described [27], JM2 significantly decreased GSC viability as observed above, but the combination of TMZ with JM2 did not decrease GSC survival further (Fig. 5C). These results were complemented by measuring caspase 3/7 activity as an indicator of apoptosis induction. We observed increasing doses of TMZ or JM2-scrambled were inefficient in inducing apoptosis in VTC-034 GSCs, whilst JM2 increased caspase 3/7 activity in these cells in a dose-dependent manner (Fig. 5D). No effect on caspase 3/7 was observed in normal human astrocytes when treated with JM2 (Fig. 5E).

**Fig. 6:**
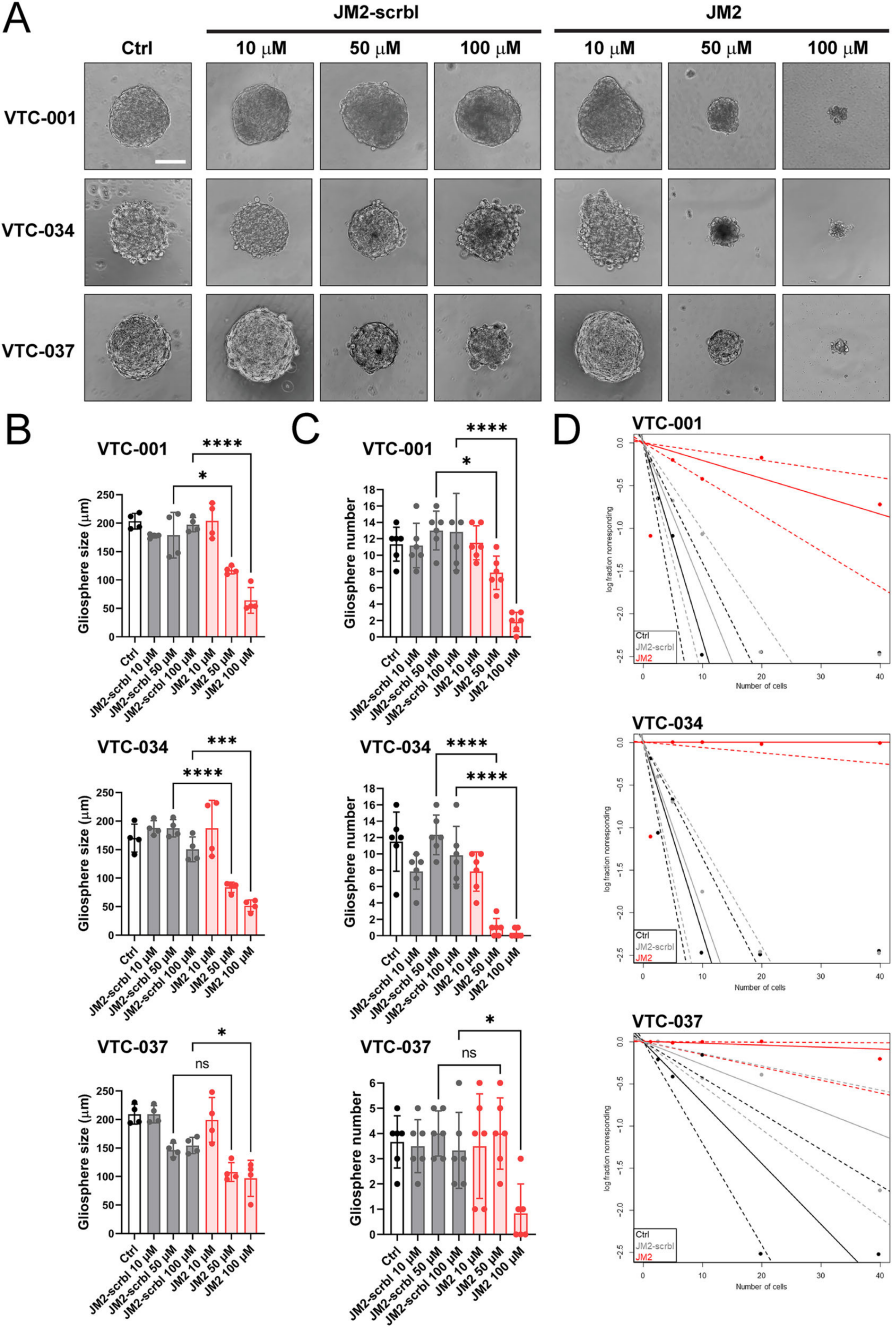
JM2 inhibits GSC-dependent gliosphere formation and self-renewal. **A** VTC-001, VTC-034, and VTC-037 GSCs were cultured as single cells in suspension in low attachment 96-well plates, and treated or not with 10, 50 or 100 μM of JM2-scrambled (JM2-scrbl) or JM2 peptides every other day for 2 weeks. Gliospheres were observed by phase-contrast microscopy. Scale bar: 100 μm. **B** Quantification of gliosphere size in (**A**) as assessed by diameter measurement (*n* = 4). **C** Quantification of gliosphere numbers in (**A**) (*n* = 6). Statistical analysis in (**B**, **C**) was performed with one-way analysis of variance (ANOVA) with Tukey’s multiple comparisons test. *p ≤ 0.05, ***p < 0.001, ****p < 0.0001, ns not significant. Data are represented as mean ± SD. **D** VTC-001, VTC-034, and VTC-037 GSCs were cultured at varying densities as single cells in suspension in low attachment 96-well plates for limiting dilution analysis, and treated or not with 100 μM of JM2-scrambled (JM2-scrbl) or JM2 peptides every other day for 2 weeks. Gliosphere-positive wells were determined after observation by phase-contrast microscopy, and GSC frequency was analyzed and plotted using the ELDA (Extreme Limiting Dilution Analysis) software. Statistical analysis: pairwise tests for differences in stem cell frequencies (VTC-001: *p* = 3.33e-09 for Ctrl vs JM2, and *p* = 1.37e-07 for JM2-scrbl vs JM2; VTC-034: *p* = 1.23e-17 for Ctrl vs JM2, and *p* = 4.84e-17 for JM2-scrbl vs JM2; VTC-037: 1.61e-07 for Ctrl vs JM2, and *p* = 0.00141 for JM2-scrbl vs JM2).

We next evaluated the effect of JM2 on GSC-dependent gliosphere formation, as an additional measure of GSC maintenance and survival. We found that JM2 inhibits VTC-001, VTC-034, and VTC-037 GSC-dependent gliosphere formation in a dose-dependent manner (Fig. 6A), with a concomitant significant decrease in gliosphere size as assessed by diameter measurement (Fig. 6B), and number (Fig. 6C) following treatment. To determine whether JM2 affects GSC self-renewal abilities, we analyzed the effect of JM2 on VTC-001, VTC-034, and VTC-037 GSCs using limiting dilution analyses. We observed JM2 significantly inhibits GSC self-renewal potential as shown with a significant decrease in VTC-001, VTC-034, and VTC-037 GSC frequency compared to JM2-scrambled or control (Fig. 6D).

### JM2 inhibits Notch signaling

**Fig. 7:**
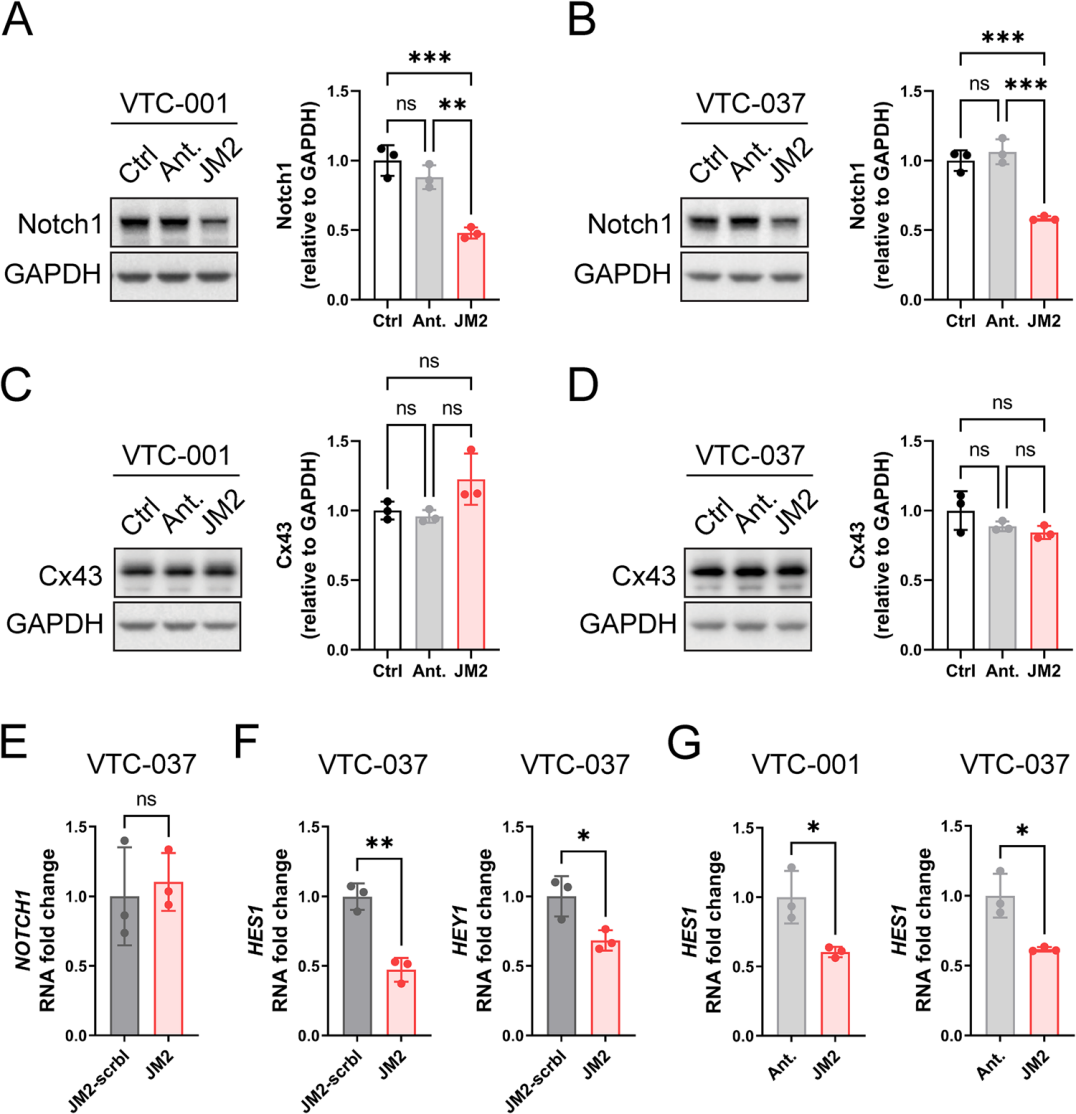
JM2 inhibits Notch signaling in GSCs. VTC-001 and VTC-037 GSCs were treated or not with antennapedia (Ant.) or JM2 peptides at 50 μM for 24 h. Cell lysates were analyzed by immunoblotting using antibodies against Notch1 (**A**, **B**; quantification shown on right), Cx43 (**C**, **D**; quantification shown on right), and GAPDH as loading control. Statistical analysis was performed with one-way analysis of variance (ANOVA) with Tukey’s multiple comparisons test. **p ≤ 0.01, ***p ≤ 0.001, ns not significant. Data are represented as mean ± SD. **E** VTC-037 GSCs were treated or not with JM2-scrambled (JM2-scrbl) or JM2 peptides at 50 μM for 24 h, RNA was extracted and *Notch1* mRNA levels were quantified by RT-qPCR. **F** VTC-037 GSCs were treated or not with JM2-scrambled (JM2-scrbl) or JM2 peptides at 50 μM for 24 h, RNA was extracted and *HES1* and *HEY1* mRNA levels were quantified by RT-qPCR. **G** VTC-001 and VTC-037 GSCs were treated or not with antennapedia (Ant.) or JM2 peptides at 50 μM for 24 h, RNA was extracted and *HES1* mRNA levels were quantified by RT-qPCR. Statistical analysis in (**E**−**G**) was performed by two-tailed unpaired Student’s *t*-test; *p ≤ 0.05, **p ≤ 0.01, ns not significant. Data are represented as mean ± SD.

Notch signaling plays a critical role in GSC survival, self-renewal capacity, and chemoresistance [17, 18, 20]. As we observed JM2 significantly decreases TMZ-resistant GSC viability and self-renewal, we tested the effect of JM2 on Notch1 expression and downstream signaling targets in GSCs. JM2 significantly decreased Notch1 expression at the protein level after 24 h of treatment in VTC-001 and VTC-037 GSCs (Fig. 7A, B), without affecting Cx43 expression (Fig. 7C, D). Interestingly, Notch1 expression was not affected at the transcription level in VTC-037 GSCs (Fig. 7E). Activation of Notch signaling results in Notch cleavage and intracellular Notch translocation to the nucleus to activate the transcription of downstream targets, including Hes1 and Hey1 [22]. We performed RT-qPCR and confirmed JM2 significantly decreased the expression of Notch downstream targets Hes1 and Hey1 at the transcription level compared to antennapedia alone or JM2-scrambled in VTC-001 and VTC-037 GSCs (Fig. 7F, G).

### JM2 decreases human GSC-derived and GBM patient-derived xenograft tumor growth in vivo

To test the effect of JM2 on GSCs in vivo, we utilized previously characterized GSCs isolated and enriched from human GBM LN229 cells (LN229/GSCs). These cells express Cx43 and form tumors following injection in mouse flank [27]. We first assessed the effect of JM2 on LN229/GSCs in vitro, and confirmed JM2 significantly decreased LN229/GSC survival after 4 days, similar to the results obtained with other GSC cell lines in this study (Supplementary Fig. 3A). Next, we injected LN229/GSCs in mouse flanks, and upon tumor formation 7 days later, we administered JM2 intratumorally every other day (Supplementary Fig. 3B). We observed a significant decrease in tumor growth in mice treated with JM2 compared to control or JM2-scrambled after 33 days (Supplementary Fig. 3C). Tumor sections were stained and analyzed for cell death using TUNEL, and we observed increased LN229-derived GSC death that positively correlated with the presence of JM2 within the tumors, while no significant cell death was observed in the control or JM2-scrambled groups (Supplementary Fig. 3D). These results were further confirmed by H&E staining (Supplementary Fig. 3E).

**Fig. 8:**
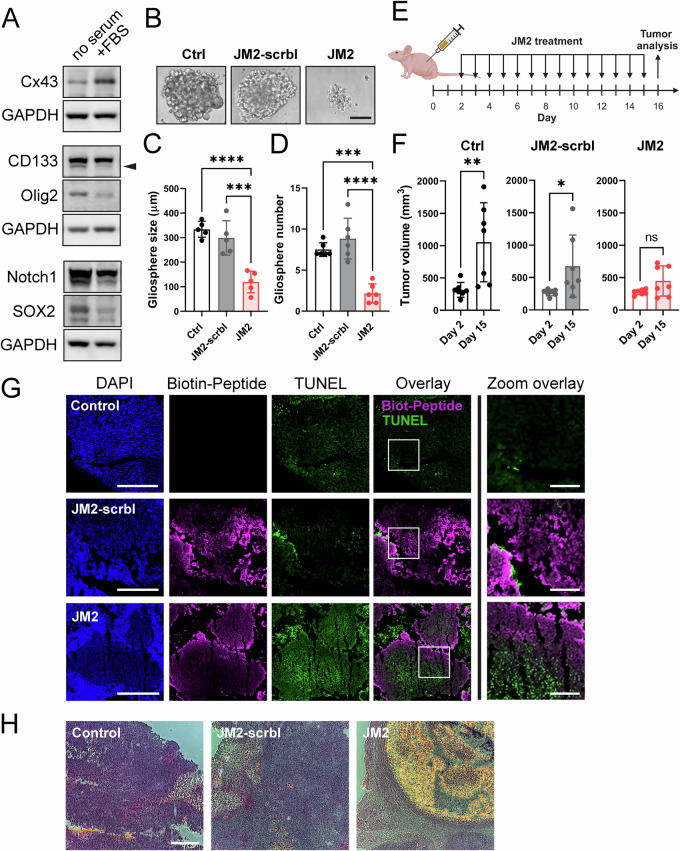
JM2 inhibits patient-derived xenograft tumor growth in vivo. **A** Following isolation from mouse flank, patient-derived xenograft (PDX) GBM22 cells were cultured as gliospheres or differentiated after addition of 10% FBS ( + FBS) for 24 h, lysed, and analyzed by immunoblotting using antibodies against Cx43, CD133, Olig2, Notch1, SOX2, and GAPDH as loading control. **B** PDX GBM22 cells were cultured as single cells in suspension in low attachment 96-well plates, and treated or not with 100 μM of JM2-scrambled (JM2-scrbl) or JM2 peptides every other day for 2 weeks. Gliospheres were observed by phase-contrast microscopy. Scale bar: 100 μm. **C** Quantification of gliosphere size in (**B**) as assessed by diameter measurement (*n* = 5). **D** Quantification of gliosphere numbers in (**B**) (*n* = 6). Statistical analysis in (**C**, **D**) was performed with one-way analysis of variance (ANOVA) with Tukey’s multiple comparisons test. ***p < 0.001, ****p < 0.0001. Data are represented as mean ± SD. **E** PDX GBM22 were injected in mouse flank, and after 2 days, vehicle/control, JM2-scrambled (JM2-scrbl) or JM2 at 300 μM were administered intratumorally every day for 14 days (created with BioRender.com). **F** Tumor volume were determined at day 2 before the first administration, and day 15 before the last administration (*n* = 7 for each treatment group). A two-tailed unpaired Student’s *t*-test was used; *p ≤ 0.05, **p ≤ 0.01, ns not significant. Data are represented as mean ± SD. **G** After 16 days, tumors were harvested and tumor sections were analyzed for the presence of biotin-tagged JM2-scrambled (JM2-scrbl) and JM2 using streptavidin-conjugated to Alexa Fluor 647 (magenta), and cell death was assessed using TUNEL staining (green). DAPI staining was used to detect nuclei (Scale bar: 500 μm). Zoomed in image from white squares on right (Scale bar: 125 μm). **H** Hematoxylin and Eosin staining of tumor sections in (**G**). (Scale bar: 500 μm).

We then evaluated the effect of JM2 on tumor growth in vivo using patient-derived xenograft (PDX) GBM22 cells. This PDX model was previously established from human GBM biopsies, and only maintained and propagated in vivo by ectopic implantation into the flanks of athymic nude mice [39]. Therefore, these xenografts retain many patient-specific characteristics such as their intrinsic gene and protein expression [43]. Similar to what we observed in VTC-001, VTC-034, and VTC-037 GSCs in Fig. 1, CD133, Olig2, SOX2, and Notch1 expression levels are higher in GBM22-derived gliospheres compared to their FBS-treated counterparts, while Cx43 expression increases following addition of FBS for 24 h (Fig. 8A). We then assessed the effect of JM2 on GBM22 cell survival using the gliosphere formation assay (Fig. 8B). We confirm JM2 significantly decreases GBM22-derived gliosphere size as assessed by diameter measurement (Fig. 8C), and number (Fig. 8D) following treatment. Next, we injected GBM22 cells in mouse flanks, and after 48 h, we administered JM2, JM2-scrambled or vehicle/control intratumorally every day (Fig. 8E). While we observed GBM22-derived tumor formation in the control and JM2-scrambled groups, we did not detect any significant tumor growth in JM2-treated animals after 14 days of treatment (Fig. 8F). After harvesting the tumors, tumor sections were stained and analyzed for cell death using TUNEL. Positive JM2 signal within the tumors correlated with an increase in cell death, while no significant correlation was observed in control and JM2-scrambled tumor sections (Fig. 8G). Increased cell death within JM2-treated tumors was further confirmed by H&E staining (Fig. 8H).

## Discussion

The role of Cx43 in cancer progression is dynamic and complex, with Cx43 described as both a tumor suppressor and oncogenic protein, depending on cancer type and stage [24]. This is not only due to differential expression of Cx43 during cancer progression, but is also linked to channel-dependent and -independent functions of Cx43 and its roles in dynamic protein complex formation in regulation of cell proliferation, migration/invasion, and apoptosis [24, 44, 45]. Although Cx43 has been extensively studied in GBM, parsing out specific oncogenic functions has historically been confounded by this association of Cx43 in both suppressing and promoting cancer [46, 47]. Our data help reconcile this apparent contradiction by identifying a specific function of non-channel Cx43 in GSC maintenance and survival.

Cx43 expression is highly variable in GSCs, GBM cell lines, and patient tissues [26, 27, 30, 48]. Increased levels of Cx43 correlate with TMZ resistance in GBM cells, and GBM patients that present high levels of Cx43 mRNA and low tumor levels of O^6^-methylguanine-DNA methyltransferase (MGMT), an enzyme that repairs TMZ-induced DNA lesions, have significantly shorter life spans than those with low levels of Cx43 mRNA [25–27]. In addition, brain metastatic cancer cells utilize Cx43 gap junctions to communicate with normal astrocytes to support tumor growth, invasion, and chemoresistance via, among other mechanisms, the formation of a nanotube communication networks [28]. In differentiated GBM cell lines, expression of a dominant negative Cx43 mutant that blocked gap junction and cell-cell communication was found to increase cell invasion [49]. However, Cx43 is also associated with anti-proliferative effects in glioma and reduced levels of Cx43 protein was reported in high-grade gliomas [29, 30]. Our study isolates a novel and specific cytoplasmic non-channel function of Cx43 in complexing with microtubules to promote GSC maintenance, self-renewal, and survival, independent of other channel-based/tumor suppressive Cx43 functions. These data highlight the importance of determining Cx43 subcellular localization in addition to its expression.

Although we observe variation in Cx43 expression in our three GSC lines, where VTC-034 GSCs present lowest relative Cx43 levels, the association of Cx43 with microtubules is consistent across all. STORM localization data (Fig. 2D, E; Supplementary Fig. 1) do not reflect protein abundance, and can quantify protein-protein interaction independent of such varying protein levels between cell lines. Our STORM findings confirm a consistent phenotype of Cx43/microtubule binding in all three GSC lines. Similarly, the tumorigenic aspect of this interaction is confirmed through our implementation of the JM2 peptide, where GSC viability and self-renewal is significantly reduced. Among other connexins, Cx46 has been shown to be critical for GSC survival and self-renewal, although in this case through a gap junction-associated function [33, 50]. The contribution of both Cx43 and Cx46 may therefore represent a synergistic combination of alterations in gap junction connexin isoform composition (Cx46) together with altered cytoskeletal regulation (Cx43) and associated changes to cell phenotype.

GSCs play a critical role in GBM resistance to treatment and tumor recurrence [8–11]. Proliferating GSCs display a loss of Cx43-dependent gap junctions at the plasma membrane that is accompanied by reduced intercellular communication, and Cx43 has been shown to be expressed at much lower levels in GSCs compared to their differentiated counterparts [32, 33]. Although we confirmed Cx43 expression is low in GSCs compared to differentiated GSCs, we demonstrate, for the first time, an enrichment of Cx43 non-junctional cytoplasmic localization at the microtubules in GSCs. While Cx43 is known to oligomerize at the trans-Golgi network before trafficking to the plasma membrane through vesicular transport along microtubules [31], our implementation of STORM allowed us to parse out an intracellular population of Cx43 directly interacting with microtubules independent from the cytoplasmic Cx43 hemichannels being transported in vesicles along microtubules. Localizing these molecules at nanoscale resolution supersedes previous biochemical and imaging techniques, which failed to distinguish this novel phenomenon – namely, two functionally distinct cytoplasmic populations of Cx43 intimately associated with the microtubule cytoskeleton.

Microtubules are critical in controlling key cellular processes including division, motility, differentiation, transport of cargoes and vesicles, and overall survival of cells [51]. The functions, together with the stability, and highly dynamic properties of microtubules, are regulated by post-translational modifications and interactions with microtubule binding proteins (MTBPs) [52]. MTBPs present different roles in stabilizing/destabilizing microtubules, as well as in anchoring microtubules to cytoskeletal components. Here, we identify Cx43 as a novel MTBP in GSCs, however, the exact role of Cx43 in regulating microtubule function, as well as the precise subcellular compartment within which Cx43 interacts with microtubules, remain to be elucidated. In addition to modulating microtubule dynamics to effect vesicular transport and/or cell membrane and junctional remodeling, Cx43 complexing with tubulin may compete with signaling molecules to elicit activation/inactivation of pathways regulating survival and differentiation, for example.

The tubulin-interacting domain within the ~130 amino acid CT of Cx43 has been well characterized [34, 53]. One study identified residues ^239^RV^240^ and ^247^YHAT^250^ to be critical for Cx43 interaction with microtubules [54]. Furthermore, Src phosphorylation of Cx43 on Y^247^ has been shown to disrupt this interaction, suggesting a dynamic process of Cx43 interaction with microtubules [54]. The Cx43 interaction domain on tubulin, however, has not been well characterized. Using in vitro recombinant proteins, it has been reported that the domain that comprises residues 114-243 on β-tubulin is necessary for Cx43 to interact with microtubules [55]. Moreover, differential expression of tubulin in GSCs has been speculated to impact resistance to chemotherapeutics, many of which historically target microtubules [56]. Our findings therefore indicate dynamic complexing of Cx43 with tubulin can be modulated by the cell and enhanced to maintain specific states advantageous to GBM progression, including in the maintenance of GSCs.

Using JM2, a Cx43 mimetic peptide of Cx43 tubulin-binding domain, we confirm efficient and specific disruption of Cx43-microtubule interaction in GSCs together with decreased self-renewal abilities and enhanced killing of this cancer stem-like cell population, highlighting a novel tumorigenic role for Cx43 in GSCs, and GBM. Thermal shift assays reveal JM2 presents high affinity for both α- and β- tubulin. While chemotherapeutic drugs such as paclitaxel have been used to target microtubule functions as therapeutic agents, many of these therapeutic agents cause significant side-effects [57]. Moreover, slower replicating cancer cells, including GSCs, are resistant to such strategies [58]. Here we highlight a novel strategy targeting Cx43/microtubule complexing alone, and presumably maintaining other roles of the cytoskeleton in normal tissues. It is possible that the Cx43 sub-population we identify as directly bound to microtubules is in the endoplasmic reticulum, potentially providing anchoring and stabilizing cytoskeletal ‘highways’ within the GSC that are disorganized by JM2. Prior work with JM2 indicated a loss of Cx43 delivery to the cell surface due to increased Cx43/tubulin complexing, but this work did not attempt to isolate vesicular Cx43 *vs* that directly bound to microtubules [41]. Additionally, the cellular context of primary GSCs (which have few gap junctions to begin with) further highlights the specificity of this biology to such cells.

Mimetic peptides of Cx43 have been implemented to efficiently modulate Cx43 channel-dependent and -independent functions [59]. The Cx43 mimetic peptide of the ZO-1 binding domain aCT1 that selectively inhibits Cx43 hemichannel activity and increases Cx43 dependent gap junction formation [60, 61], restores TMZ sensitivity to TMZ-resistant GBM cells [27]. A Cx43 mimetic peptide of a short region within Cx43 C-terminus that recruits and inhibits c-Src activity, decreases cell motility, invasion, and proliferation in GSCs [62]. Peptide-based cancer therapies have recently gained increasing recognition and validation in the field. In fact, peptides present several advantages when compared to small molecules and kinase inhibitors with peptides demonstrating higher specificity in targeting cancer cells and/or their associated tumorigenic aspects, and exerting lower toxicity in normal tissues [63]. Importantly, although astrocytes express high level of Cx43, we observed no toxic effect of JM2 on normal human astrocytes in vitro, suggesting that Cx43/microtubule complex formation is cell-type specific in its putative role in GSC maintenance.

While several signaling pathways have been involved in GSC maintenance such as TGF-β, Hedgehog-GLI1 or Wnt/β-catenin [19, 64–66], Notch signaling emerges as a critical pathway in the regulation of GSC self-renewal [17, 19, 20, 67]. As we observed JM2 can significantly inhibit self-renewal capacity of GSCs using limiting dilution assays, we investigated the effect of JM2 on Notch signaling. We demonstrated that JM2 significantly decreased Notch expression and downstream signaling in GSCs, wherein we determined downregulation of *HES1* and *HEY1* transcription (Fig. 7). Activation of the Notch pathway is associated with GSC proliferation, self-renewal, survival, and maintenance of stemness in GSCs [15, 17]. Cell-cell contacts lead to interaction and activation of Notch receptors by Delta-like or Jagged ligands on apposing cells, prior to two enzymatic cleavages that promote internalization and nuclear translocation of the Notch intracellular domain (NICD). There, NICD regulates the transcription of genes such as *HES1* and *HEY1*, belonging to the basic helix-loop-helix (bHLH) family of transcription factors, which in turn, regulate the expression of genes involved in the maintenance of stem-like properties in GSCs [17]. The vascular niche of GBM tumors has been shown to sustain GSC survival [68]. Given the major role of Notch signaling in the vasculature, high levels of Delta-like/Jagged ligands likely maintain GSCs in these niches. While we do not know the exact mechanism of JM2-mediated Notch pathway disruption, alterations in microtubule trafficking likely dull the ability of GSCs to engage with and respond to ligands, compromising survival. Indeed, through high specificity of a peptide therapeutic drug, in our case JM2 binding to microtubules, we speculate the effect of JM2 on Notch signaling may result from displacing Cx43 away from microtubules and/or affecting microtubule dynamics. Further analysis of JM2 effect on Notch signaling or other pathways that promote GSC maintenance and self-renewal remain to be explored.

Given the lack of progress in treatment of GBM, new approaches in targeting not just GBM cancer cells, but also GSC populations likely persisting after surgical resection of tumors and following treatments, are critical. Based on our preclinical findings, administration of JM2 in combination with chemotherapy (TMZ) may improve patient survival through slowing of tumor recurrence. The therapeutic potential of JM2 is confirmed in our in vivo results where we observe a significant inhibition of GBM tumor growth after inoculation of a human PDX model enriched with GSC markers or LN229-enriched GSCs in mouse flank. Importantly, we did not observe any effect on Cx43 expression in JM2-treated GSCs. By targeting a specific function of Cx43, and not gap junctions (which are essential in many organ systems), Cx43 expression, or tubulin polymerization (e.g., Taxol), we anticipate fewer side effects based on our data in normal astrocytes, for example. While a promising class of drugs, peptides do present their challenges in the context of administration and low stability in vivo. Delivery systems such as nanoparticles show some potential here [69]. Extracellular vesicles (EVs) are also emerging as a promising and specific means of delivery payloads such as peptide drugs to specific cell-types, including tumors [70, 71]. Together, our work identifies Cx43/tubulin complexing as a specific event in GSCs, and represents a step forward in clarifying oncogenic *vs* tumor suppressive roles for connexins in GBM. The therapeutic approach we posit, and provide data for, maintains the homeostatic functions of gap junctions, whilst selectively targeting Cx43/tubulin complexes with downstream perturbation of Notch signaling pathways required for GSC survival. In sum, JM2 presents a mechanistically intriguing and promising new GBM therapeutic for treating this devastating disease.

## Supplementary information

Supplemental Figures

Full-length western blots

## Data Availability

All data generated and/or analyzed during this current study are included in this published article and its supplementary information files, and/or available from the corresponding authors on reasonable request.
