# Supplementary material for: Cytoplasmic connexin43-microtubule interactions promote glioblastoma stem-like cell maintenance and tumorigenicity
Source: Cell Death Dis. 2025 May 16;16(1):388. doi: 10.1038/s41419-025-07514-2 (PMC12084297; doi:10.1038/s41419-025-07514-2)
Supplement: Supplementary file 1 — Supplemental Figures [file 41419_2025_7514_MOESM1_ESM.docx]

**Cytoplasmic connexin43-microtubule interactions promote glioblastoma stem-like cell maintenance and tumorigenicity.**

James W. Smyth, Sujuan Guo, Lata Chaunsali, Laurie O’Rourke, Jacob Dahlka, Stacie Deaver, Michael Lunski, Elmar Nurmemmedov, Harald Sontheimer, Zhi Sheng, Robert G. Gourdie and Samy Lamouille

**Supplemental Figures**

**
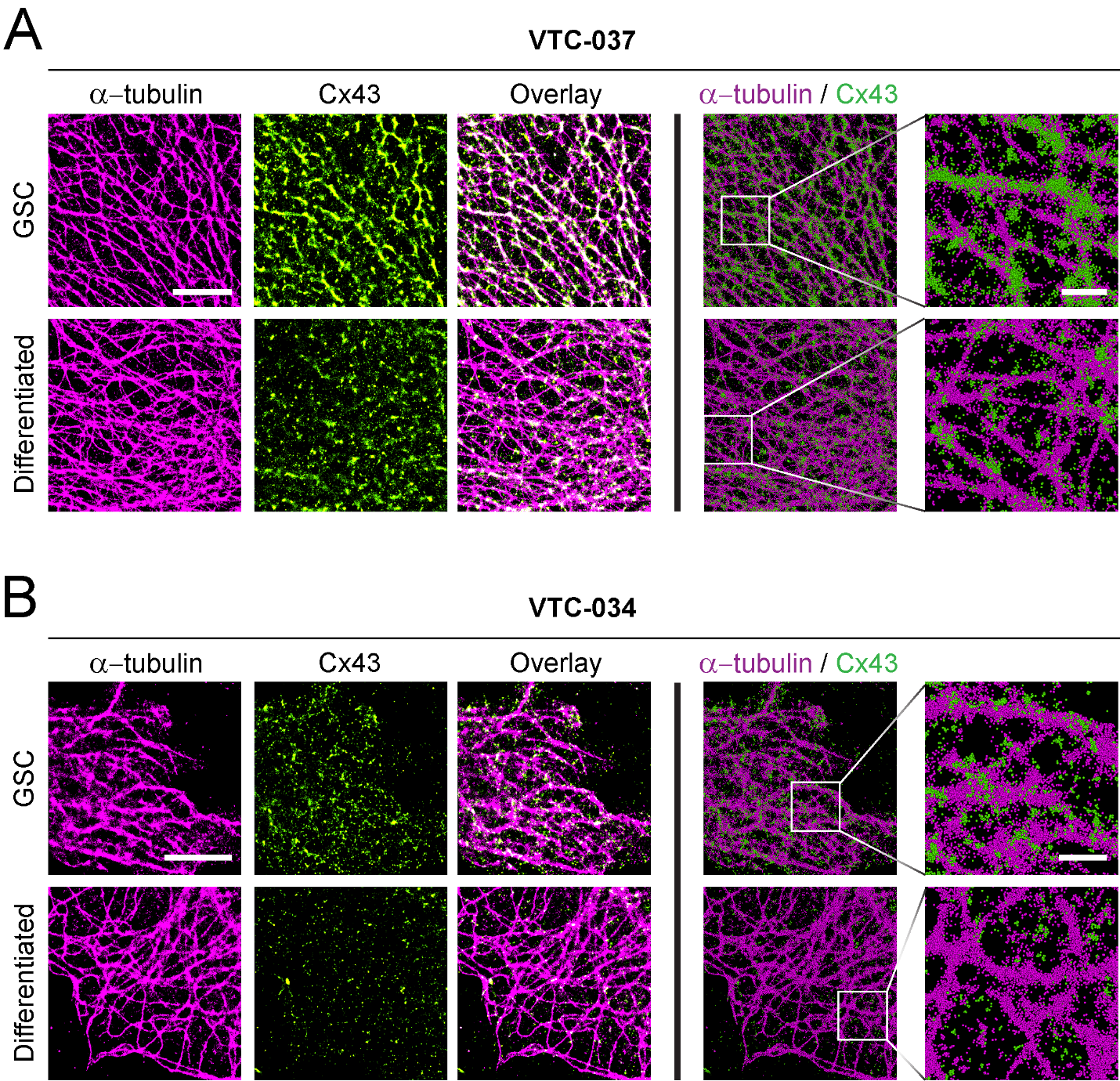
**

**Supplemental Figure 1: Increased Cx43 interaction with microtubules in GSCs.** Stochastic optical reconstruction microscopy (STORM) derived localizations of Cx43 (green) and α-tubulin (magenta) in VTC-037 (**A**) and VTC-034 (**B**) GSCs or differentiated by addition of 10% FBS for 24 h. Left 6 panels: point-splatting visualization of STORM localizations to better identify complexing (white; scale bar: 5 μm). Right 4 panels: point-clouds of 50 nm spheres representing individual localizations, including zoomed-in regions (scale bar: 1 μm).


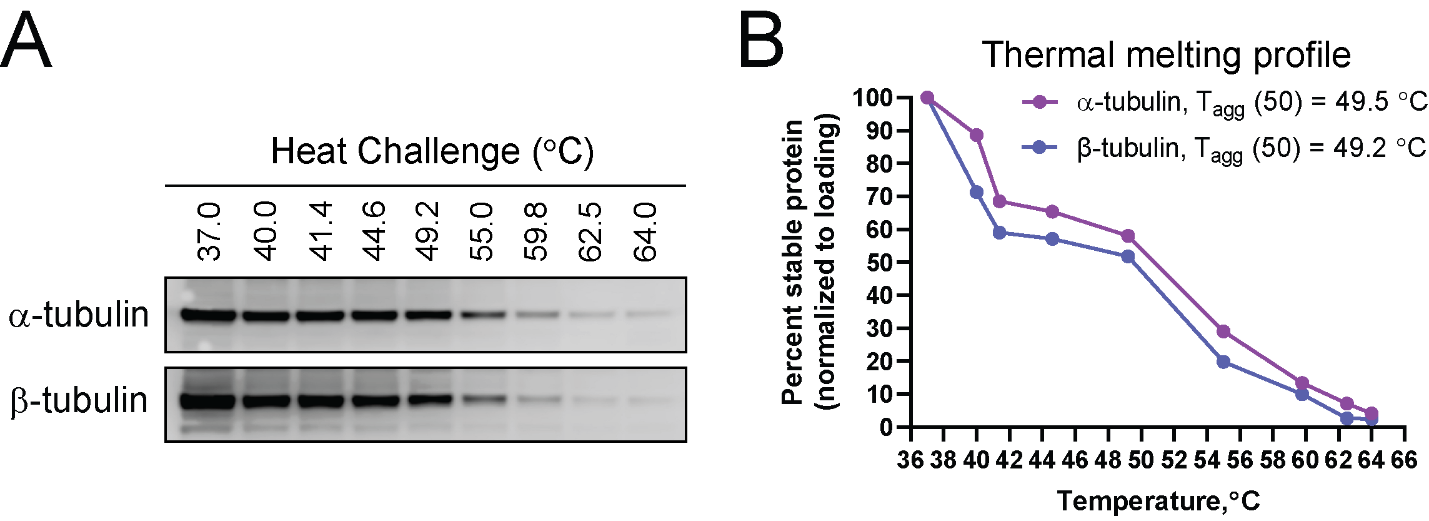


**Supplemental Figure 2: Cellular thermal shift assay in VTC-037 GSC lysates.** Heat gradient was used on VTC-037 GSC lysates to determine thermal melting profiles of α-tubulin and β-tubulin, analyzed by immunoblotting (**A**), and represented as percent stable protein over increased temperatures (**B**).

**
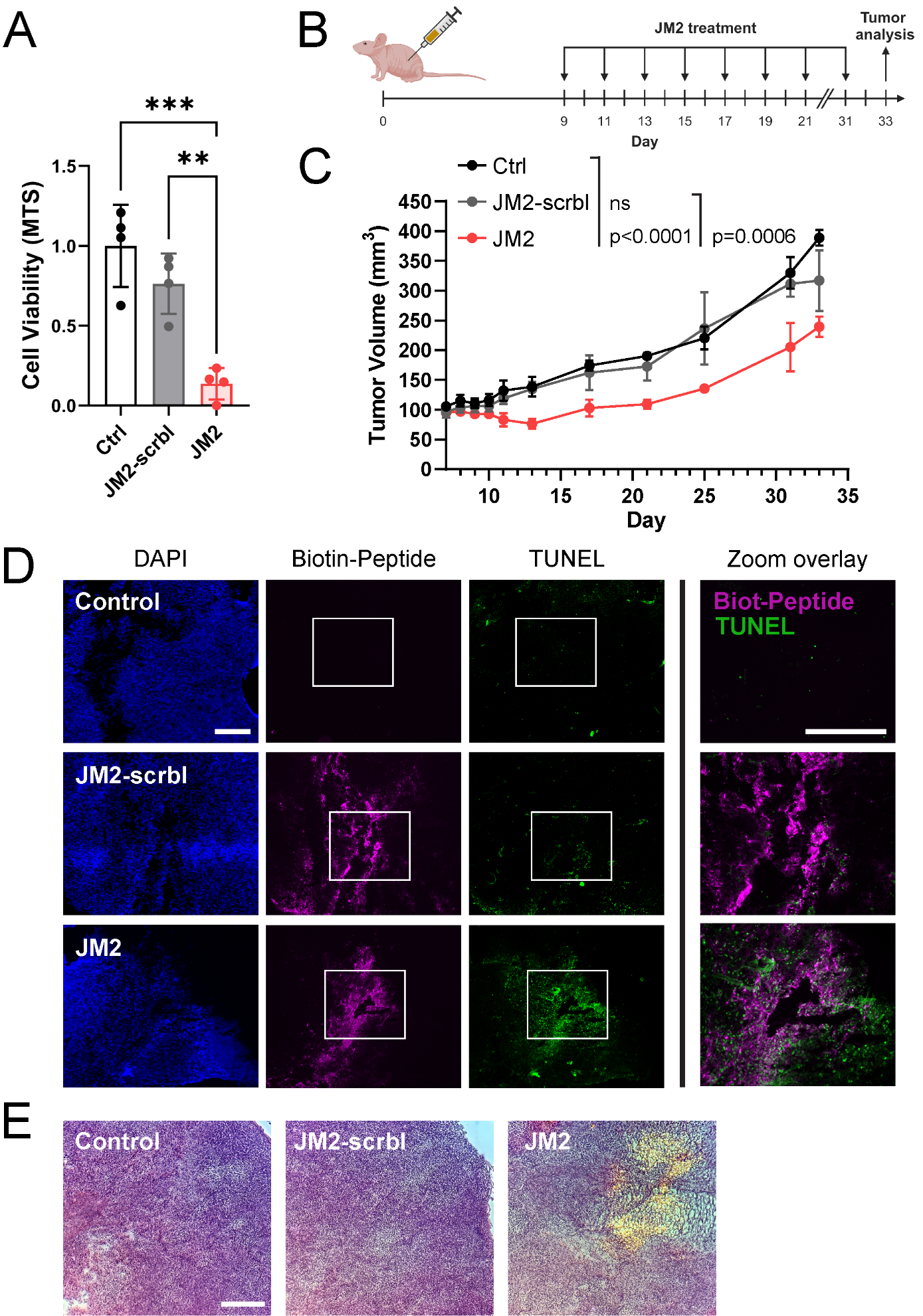
**

**Supplemental Figure 3: JM2 inhibits GSC survival *in vivo*. A)** LN229/GSCs were treated or not with 100 μM of JM2-scrambled (JM2-scrbl) or JM2 peptides for 4 days before cell survival was assessed using MTS assay. Statistical analysis was performed with one-way analysis of variance (ANOVA) with Tukey’s multiple comparisons test. ***p*≤0.01, ****p*≤0.001. Data are represented as mean ± SD. **B)** LN229/GSCs were injected in mouse flank and upon tumor formation, JM2-scrambled (JM2-scrbl) or JM2 at 300 μM were administered intratumorally every other day (created with BioRender.com). **C)** Tumor volume was determined at different time points (n=3 for each treatment group). The rate of tumor growth over the first 31 days was different among all three interventions (X2(2)=28.1845; p<0.0001). Specifically, the rate of tumor growth in JM2 was significantly slower than that of the Control (t(87)=-5.081; p<0.0001) and JM2-scrambled (t(87)=-3.873; p=0.0006). Data are represented as mean ± SEM. **D)** After 33 days, tumor sections from C were analyzed for the presence of biotin-tagged JM2-scrambled (JM2-scrbl) and JM2 using streptavidin-conjugated to Alexa Fluor 647 (magenta), and cell death was assessed using TUNEL staining (green). DAPI staining was used to detect nuclei (Scale bar: 400 μm). Zoomed in overlay image from white squares on right. **E)** Hematoxylin and Eosin staining of tumor sections in D (Scale bar: 500 μm).
