# Supplementary material for: Cytoplasmic connexin43-microtubule interactions promote glioblastoma stem-like cell maintenance and tumorigenicity
Source: Cell Death Dis. 2025 May 16;16(1):388. doi: 10.1038/s41419-025-07514-2 (PMC12084297; doi:10.1038/s41419-025-07514-2)

**Cytoplasmic connexin43-microtubule interactions promote glioblastoma stem-like cell maintenance and tumorigenicity.**

James W. Smyth, Sujuan Guo, Lata Chaunsali, Laurie O’Rourke, Jacob Dahlka, Stacie Deaver, Michael Lunski, Elmar Nurmemmedov, Harald Sontheimer, Zhi Sheng, Robert G. Gourdie and Samy Lamouille

**Full length western blots**


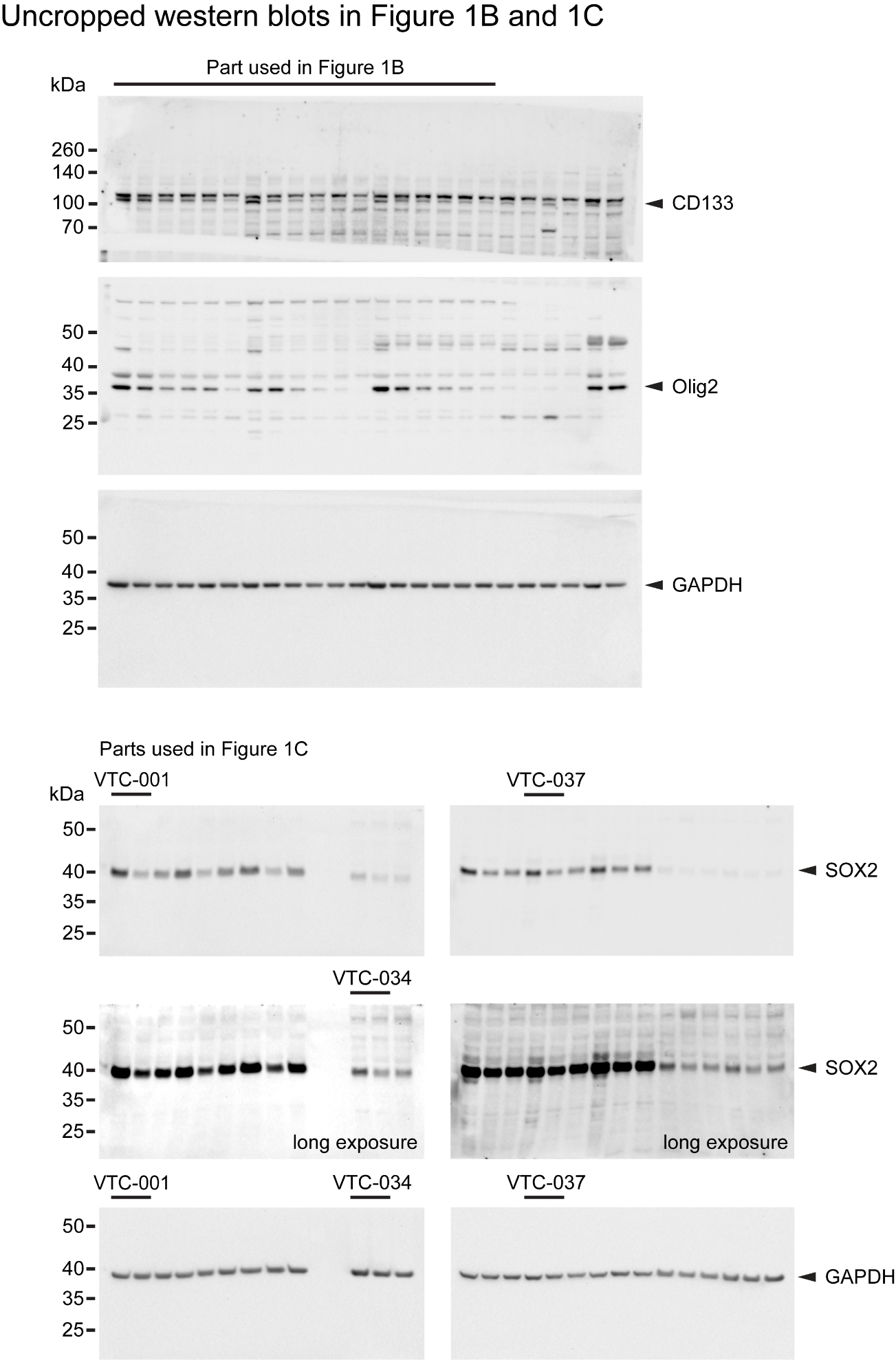


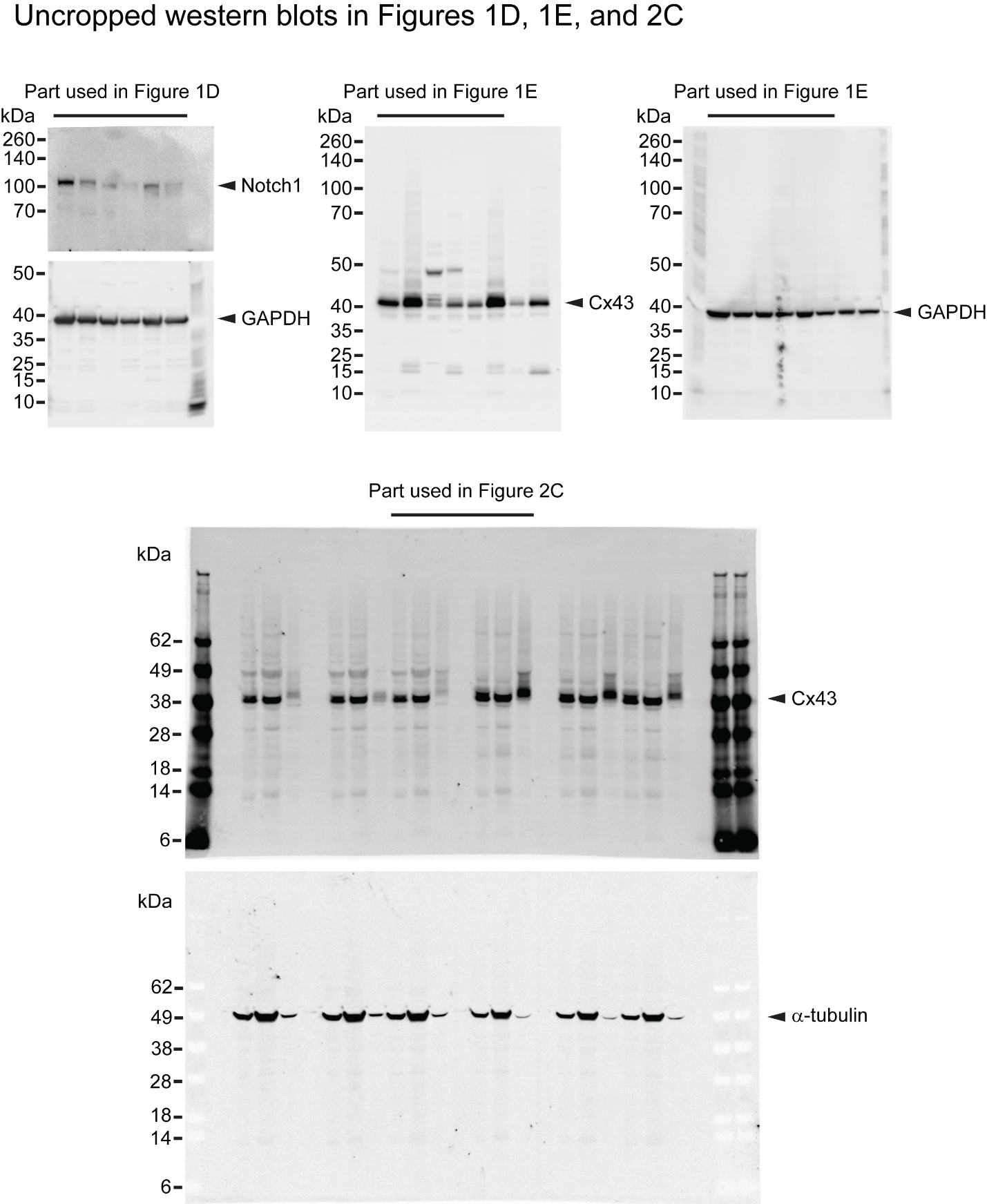


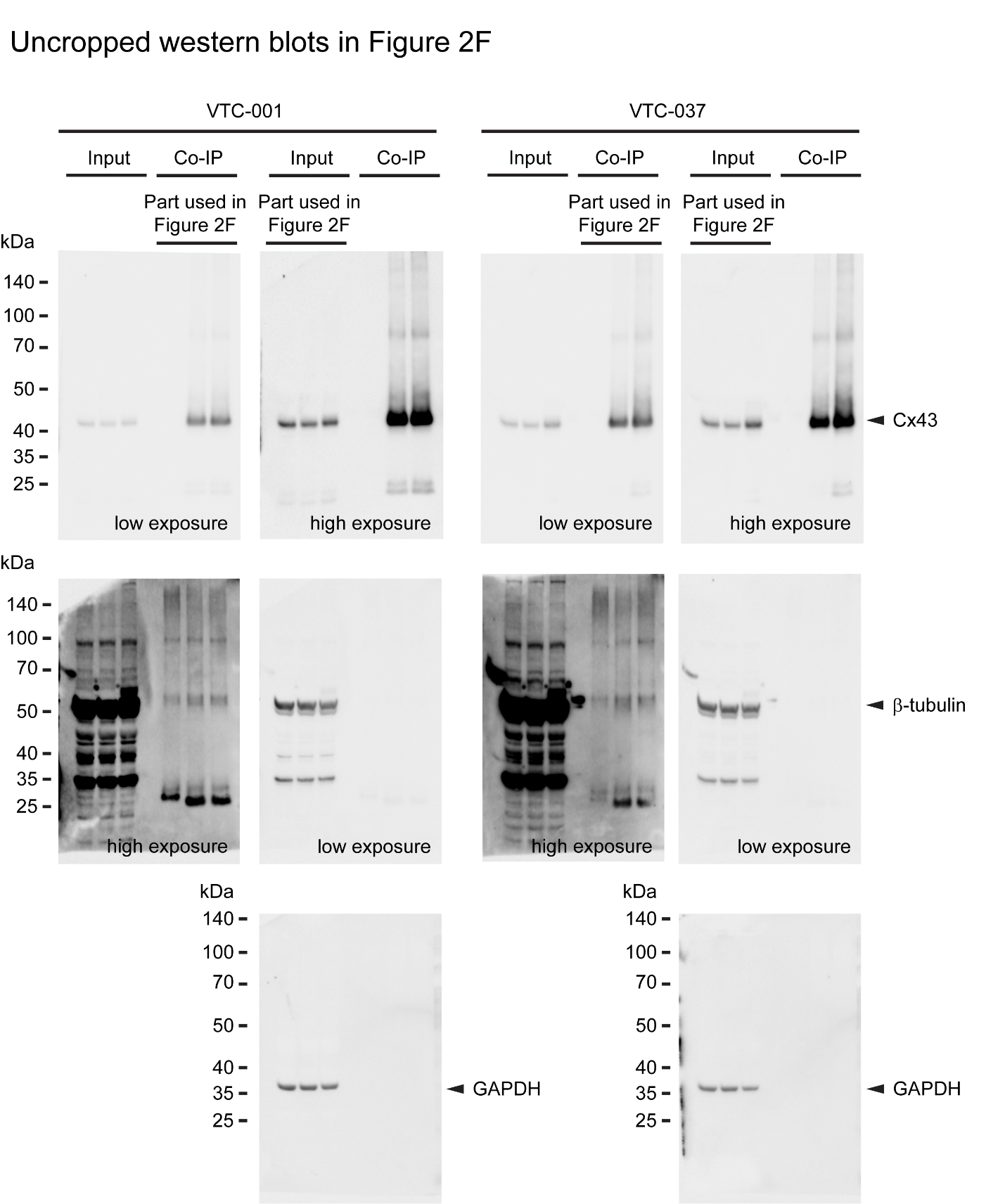


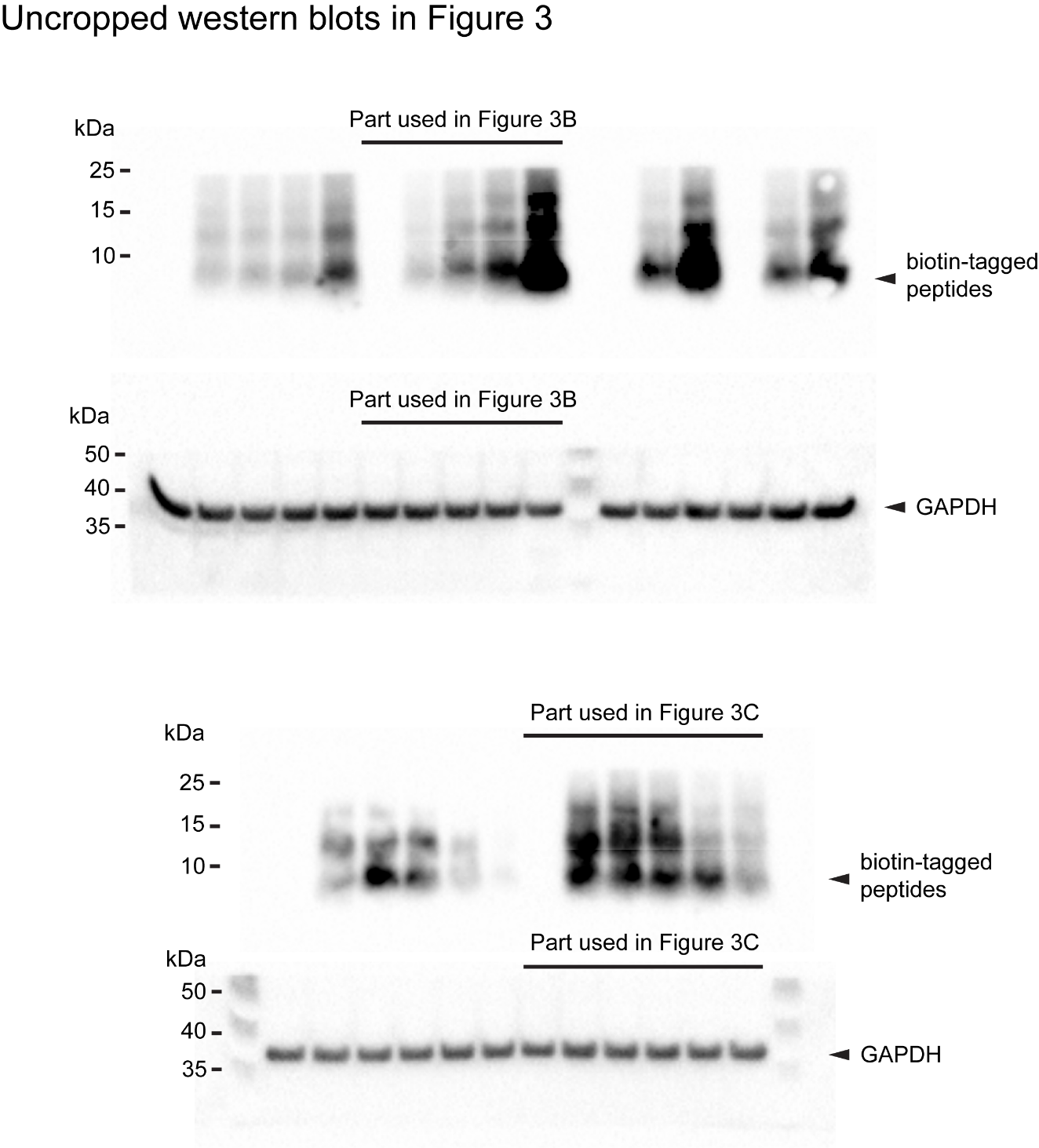


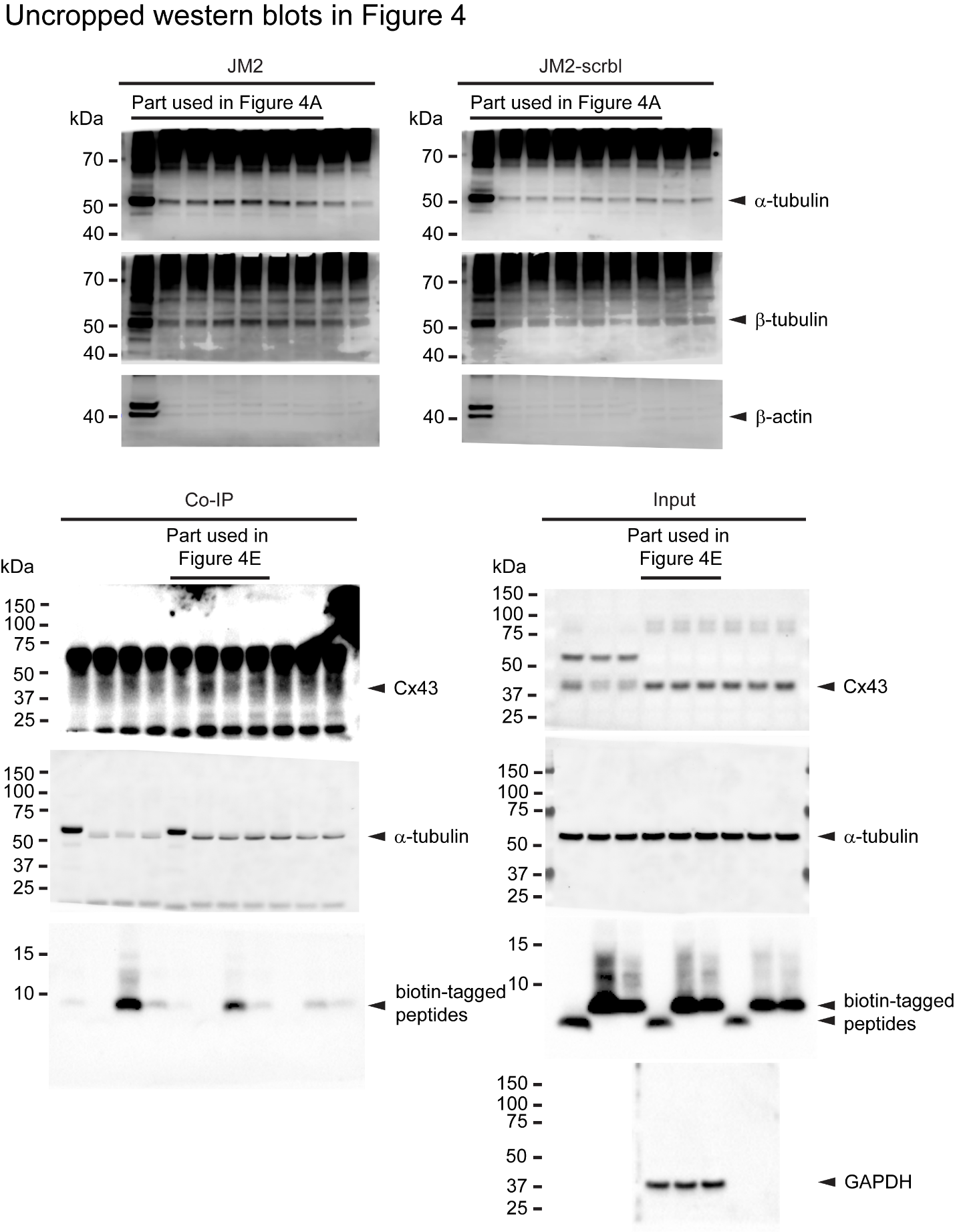


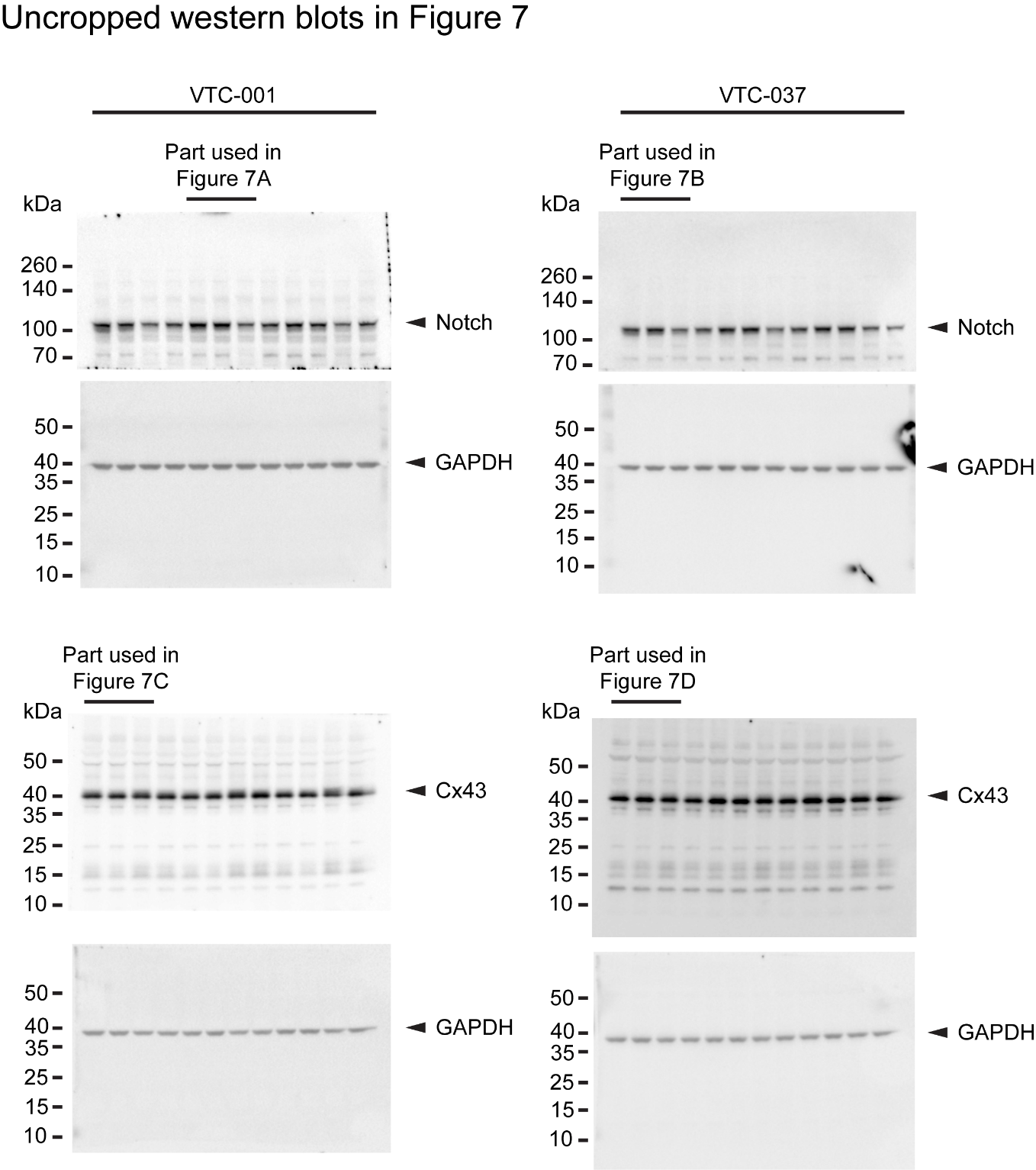
–


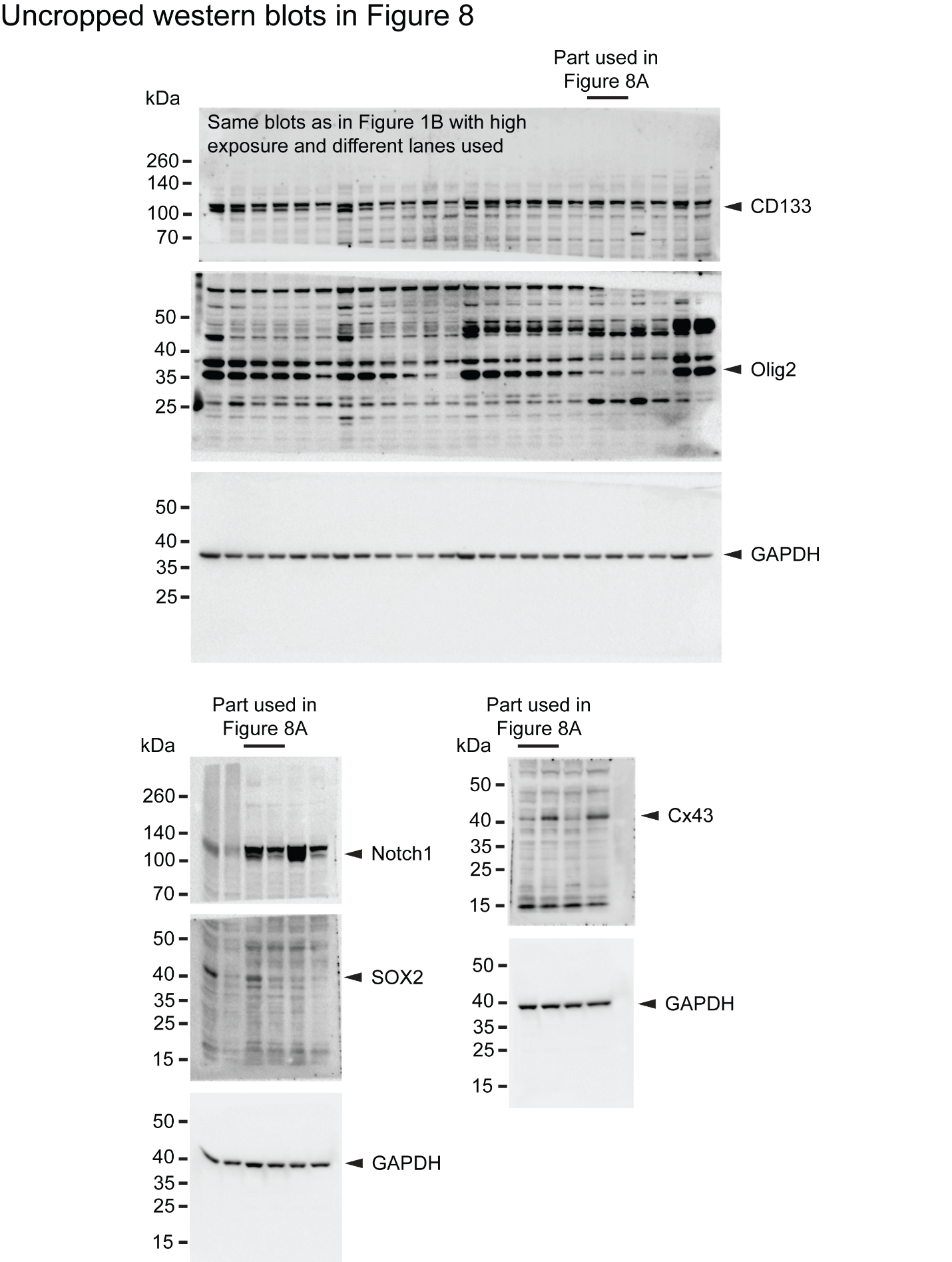

Supplement: Supplementary file 2 — Full-length western blots [file 41419_2025_7514_MOESM2_ESM.docx]
